# Hospital-Level NICU Capacity, Utilization, and 30-Day Outcomes in Texas

**DOI:** 10.1001/jamanetworkopen.2023.55982

**Published:** 2024-02-14

**Authors:** David C. Goodman, Patrick Stuchlik, Cecilia Ganduglia-Cazaban, Jon E. Tyson, JoAnna Leyenaar, Elenir B. C. Avritscher, Mathew Rysavy, Kanekal S. Gautham, David Lynch, Therese A. Stukel

**Affiliations:** 1 The Dartmouth Institute for Health Policy and Clinical Practice, Department of Pediatrics, Geisel School of Medicine at Dartmouth, Hanover, New Hampshire; 2 The Children’s Hospital at Dartmouth, Lebanon, New Hampshire; 3 Center for Health Care Data and Department of Management, Policy, and Community Health, School of Public Health, The University of Texas Health Science Center at Houston; 4 Institute for Clinical Research and Learning Health Care, McGovern Medical School at The University of Texas Health Science Center at Houston; 5 Division of Neonatology, Department of Pediatrics, Nemours Children’s Health, Orlando, Florida; 6 The University of Texas at Austin; 7 ICES, Toronto, Ontario, Canada; 8 Institute of Health Policy, Management and Evaluation, University of Toronto, Ontario, Canada

## Abstract

**Importance:**

Risk-adjusted neonatal intensive care unit (NICU) utilization and outcomes vary markedly across regions and hospitals. The causes of this variation are poorly understood.

**Objective:**

To assess the association of hospital-level NICU bed capacity with utilization and outcomes in newborn cohorts with differing levels of health risk.

**Design, Setting, and Participants:**

This population-based retrospective cohort study included all Medicaid-insured live births in Texas from 2010 to 2014 using linked vital records and maternal and newborn claims data. Participants were Medicaid-insured singleton live births (LBs) with birth weights of at least 400 g and gestational ages between 22 and 44 weeks. Newborns were grouped into 3 cohorts: very low birth weight (VLBW; <1500 g), late preterm (LPT; 34-36 weeks’ gestation), and nonpreterm newborns (NPT; ≥37 weeks’ gestation). Data analysis was conducted from January 2022 to October 2023.

**Exposure:**

Hospital NICU capacity measured as reported NICU beds/100 LBs, adjusted (ie, allocated) for transfers.

**Main Outcomes and Measures:**

NICU admissions and special care days; inpatient mortality and 30-day postdischarge adverse events (ie, mortality, emergency department visit, admission, observation stay).

**Results:**

The overall cohort of 874 280 single LBs included 9938 VLBW (5054 [50.9%] female; mean [SD] birth weight, 1028.9 [289.6] g; mean [SD] gestational age, 27.6 [2.6] wk), 63 160 LPT (33 684 [53.3%] female; mean [SD] birth weight, 2664.0 [409.4] g; mean [SD] gestational age, 35.4 [0.8] wk), and 801 182 NPT (407 977 [50.9%] female; mean [SD] birth weight, 3318.7 [383.4] g; mean [SD] gestational age, 38.9 [1.0] wk) LBs. Median (IQR) NICU capacity was 0.84 (0.57-1.30) allocated beds/100 LB/year. For VLBW newborns, NICU capacity was not associated with the risk of NICU admission or number of special care days. For LPT newborns, birth in hospitals with the highest compared with the lowest category of capacity was associated with a 17% higher risk of NICU admission (adjusted risk ratio [aRR], 1.17; 95% CI, 1.01-1.33). For NPT newborns, risk of NICU admission was 55% higher (aRR, 1.55; 95% CI, 1.22-1.97) in the highest- vs the lowest-capacity hospitals. The number of special care days for LPT and NPT newborns was 21% (aRR, 1.21; 95% CI,1.08-1.36) and 37% (aRR, 1.37; 95% CI, 1.08-1.74) higher in the highest vs lowest capacity hospitals, respectively. Among LPT and NPT newborns, NICU capacity was associated with higher inpatient mortality and 30-day postdischarge adverse events.

**Conclusions and Relevance:**

In this cohort study of Medicaid-insured newborns in Texas, greater hospital NICU bed supply was associated with increased NICU utilization in newborns born LPT and NPT. Higher capacity was not associated with lower risk of adverse events. These findings raise important questions about how the NICU is used for newborns with lower risk.

## Key Points

**Question:**

Is hospital-level neonatal intensive care unit (NICU) bed supply associated with higher risk-adjusted newborn utilization and better outcomes?

**Findings:**

In this cohort study with 874 280 newborns, NICU bed supply was associated with statistically higher likelihood of NICU admission and special care days among late preterm and nonpreterm newborns, but not among very low birth weight newborns. Higher bed supply was not associated with lower inpatient mortality and 30-day postdischarge adverse events.

**Meaning:**

These findings suggest that there may be overcapacity of NICUs in some health care regions and overuse of NICU care in some newborn populations; further investigation into the benefits of additional NICU capacity expansion is warranted.

## Teaser

This cohort study assesses the association of hospital-level neonatal intensive care unit (NICU) bed capacity with utilization and outcomes in newborn cohorts with differing levels of health risk.

## Introduction

In the past 3 decades, the number of neonatal intensive care unit (NICU) beds in the United States has increased by 50%, and the number of neonatologists per 1000 live births (LBs) has more than doubled.^1^ Today, approximately 1 in 10 infants born in the United States is admitted to a NICU.^2,3,4,5^ NICU utilization, quality, and outcomes have been shown to vary substantially among hospitals and US regions.^4,6,7,8,9,10^ Little of the variation appears to be explained by patient factors,^4,7,9,11^ and variation is highest among lower-risk infants.^4,6,10^

Efforts to rationalize NICU utilization have reduced admission rates in lower-risk newborns,^5,12^ but there remains scientific uncertainty regarding what care constitutes best practice. In the absence of high-quality evidence supporting clinical decisions, the most effective intervention at the patient level and the right rate at the population level are often unknown. Furthermore, the focus on clinical decision-making often ignores system-level factors. While these are often unnoticed by clinicians, they may exert important effects on how care is provided.^13^

This study investigates the association of a system factor, NICU capacity, as measured by reported NICU beds, with NICU utilization and infant outcomes. We hypothesized that NICU capacity was associated both with higher NICU utilization and better outcomes, as measured by lower mortality and 30-day postdischarge adverse events, in 3 population-based cohorts: very low birth weight (VLBW; birth weight <1500 g), late preterm (LPT; 34-36-weeks’ gestation), and nonpreterm (NPT; ≥37 weeks’ gestation) LBs.

## Methods

### Data Sources and Study Cohorts

The Texas Medicaid newborn cohort was developed using methods previously described.^4^ Briefly, for all LBs in Texas from January 1, 2010, to December 31, 2014, and enrolled in Texas Medicaid/Children’s Health Insurance Program (1 133 441 LBs), we linked Medicaid enrollment records, birth and death certificates, and maternal and newborn facility and professional claims and encounters through the first year of life (eFigure 1 in Supplement 1).

We studied 3 mutually exclusive cohorts: (1) VLBW with birth weight between 400 and 1499 g; (2) LPT, with gestational age (GA) 34 to 36 weeks; and (3) nonpreterm (NPT) newborns (GA >37 weeks) (eFigure 1 in Supplement 1). Multiple births and newborns with birth weight less than 400 g or GA of less than 22 week or of 45 weeks or greater were excluded. To avoid erroneous GAs, we also excluded newborns with birth weights less than the 3rd or greater than the 97th percentile for GA-sex,^14^ resulting in subcohort sizes of 9938 VLBW LBs, 63 160 LPT LBs, and 801 182 NPT LBs born in hospitals with level II to IV NICUs. Newborns were assigned to the hospital of birth even if transferred (transfer status variable is included in the risk adjustment model) and observed for the entire newborn inpatient episode (NIE), beginning at birth and ending with discharge home where any readmissions occurred more than 24 hours after discharge.

The study adheres with the Strengthening the Reporting of Observational Studies in Epidemiology (STROBE) guideline for cohort studies.^15^ The project was approved and exempted from the requirement for informed consent per 45 CFR 46.116(d) by the institutional review boards of Dartmouth College, University of Texas Health Science Center at Houston, and the Texas Health and Human Services Commission.

### Hospitals and Hospital-Level Exposures

The Texas Hospital Association Annual Survey of Hospitals^16^ was used for hospitals’ total staffed NICU beds (intensive and intermediate), birth volume, NICU level, and for-profit status for each study year. *Staffed beds* are those reported by the hospital as staffed for use (ie, operational beds). Given the common usage of the term NICU for level II to IV units,^17^ we refer to all of these beds as *NICU beds*. The primary exposure was the number of allocated NICU level II to IV beds per 100 LBs (ie, Medicaid- and non-Medicaid–covered infants) by year. NICU beds per hospital were allocated^18^ to the birth hospital. Bed numbers in hospitals receiving babies from other centers were reduced by the number occupied by these transferred newborns. Similarly, bed counts in hospitals transferring babies out (typically smaller hospitals) were increased by the number occupied by these transferred (eFigure 2 in Supplement 1).

Primary analyses were limited to hospital-years with at least 1 birth insured by Medicaid and 1 reported NICU bed (NICU level ≥II) during 2010 to 2014. Hospitals without such beds were considered level I units. Presence of neonatology fellowship programs (teaching status; 8 hospitals) during 2010 to 2014 was determined by study team members and by calls to hospitals.

### Individual-Level Variables

We used previously described^4^ methods to model cohort-specific 27-day mortality using variables preceding (ie, exogenous to) newborn medical care; model coefficients were then used to estimate death probabilities for each newborn (VLBW C statistic, 0.86; LPT C statistic, 0.87; NPT C statistic, 0.78). We included additional individual-level measures in the final capacity-utilization models: presence of a (1) major procedure, (2) diagnosis, and (3) congenital anomaly associated with NICU admissions, but not necessarily mortality, as judged by study team neonatologists (M.R., J.E.T., and K.S.G.) and pediatricians (D.C.G. and J.L.) (eAppendix in Supplement 1).

### Measures of NICU Utilization and Infant Outcomes

Adjusted risk ratios (aRRs) by bed capacity were estimated for 3 utilization outcomes: (1) NICU admission, (2) number of special care days (SCDs), and (3) SCDs conditional on NICU admission.^4^ A NICU admission was defined as newborn receiving care in a level II to IV hospital with (1) at least 1 professional claim at a nonroutine level, (2) a facility claim at the highest (ie, intensive or critical) level, or (3) died in the first 5 days without a claim indicating such care. We defined *SCD* as an inpatient day with either facility or professional nonroutine level claims. We examined 2 adverse outcomes: (1) mortality during NIE and (2) a composite measure of 30-day post-discharge mortality, emergency department visit, hospital admission, or observation day.

### Statistical Analysis

We used Poisson generalized estimating equations to estimate the association between hospital-level NICU bed capacity and inpatient newborn utilization and infant outcomes, clustering by hospital–birth year. The units of analysis were individual LBs. The model was specified a priori and included covariates for mortality risk, major diagnoses, major procedures, congenital anomalies, hospital for-profit status, and teaching status. Allocated bed capacity (<0.50 beds/100 LB, 0.50 to <0.75 beds/100 LBs, 0.75 to <1.00 beds/100 LBs, 1.00 to <1.25 beds/100 LBs, 1.25 to <1.50 beds/100 LBs, 1.50 to <1.75 beds/100 LBs, 1.75 to <2.00 beds/100 LBs, ≥2.00 beds/100 LBs) and mortality risk were categorized. aRRs were estimated relative to the category with the lowest allocated bed capacity. To investigate the association of capacity with SCDs independent of NICU admission, models were repeated restricting to NICU-admitted newborns. We conducted stratified analyses for hospital characteristics known to be associated with hospital-level NICU utilization, quality, or outcomes^19,20,21^: profit and not-for-profit status, annual hospital birth volume (greater than and less than the median), and teaching status (neonatal fellowship and not). These were planned as descriptive and were not hypothesis driven. Sensitivity analyses included: (1) including level I hospitals and (2) removing hospitals with only level II units. Tests for trend were used to test the null hypothesis in all models and were calculated with bed capacity as a continuous variable. All *P* values were 2-sided with a value of less than .05 considered statistically significant. Data analysis was conducted with SAS version 9.4 (SAS Institute) from January 2022 to October 2023.

## Results

### Cohort Characteristics

Table 1 presents maternal and newborn characteristics. The overall cohort of 874 280 single LBs included 9938 VLBW (5054 [50.9%] female; mean [SD] birth weight, 1028.9 [289.6] g; mean [SD] gestational age, 27.6 [2.6] wk), 63 160 LPT (33 684 [53.3%] female; mean [SD] birth weight, 2664.0 [409.4] g; mean [SD] gestational age, 35.4 [0.8] wk), and 801 182 NPT (407 977 [50.9%] female; mean [SD] birth weight, 3318.7 [383.4] g; mean [SD] gestational age, 38.9 [1.0] wk) LBs. The cohort included 677 mothers (6.8%) of VLBW newborns, 5139 mothers (8.1%) of LPT newborns, and 67 633 mothers (8.4%) of NPT newborns with less than high school education. Congenital anomalies were reported in 1503 VLBW newborns (15.1%), 2523 LPT newborns (4.0%), and 9879 NPT newborns (1.2%). A total of 1394 VLBW newborns (14.0%), 1189 LPT newborns (1.9%), and 4291 NPT newborns (0.5%) were transferred during the NIE. Among VLBW newborns, 9469 (95.3%) were admitted to an NICU with a mean (SD) of 54.8 (49.8) SCDs and a mean (SD) length of stay of 57.4 (53.8) days. Overall, 1210 (12.2%) died during the NIE, and 1398 (14.1%) had at least one 30-day postdischarge composite adverse event. Among LPT newborns, 24 979 (39.5%) were admitted to an NICU with a mean (SD) of 4.4 (11.0) SCDs and a mean (SD) length of stay of 5.6 (12.2) days; inpatient deaths and postdischarge events occurred in 267 (0.4%) and 5857 (9.3%), respectively. A total of 52 945 newborns born NPT (6.6%) were admitted to an NICU with a mean (SD) of 0.6 (4.6) SCDs and a mean (SD) length of stay of 2.1 (5.5) days. Inpatient deaths and postdischarge events occurred in 447 (0.1%) and 53 381 (6.7%) newborns, respectively.

**Table 1.** Texas Medicaid Newborn Study Cohorts for Texas Hospitals, 2010 to 2014^a^

Characteristics	Participants, No. (%)		
	Very low birth weight singletons (<1500 g) (n = 9938)	Late preterm singletons (34-36 wk) (n = 63 160)	Nonpreterm singletons (≥37 wk) (n = 801 182)
**Maternal characteristics**			
Education			
Less than high school	677 (6.8)	5139 (8.1)	67 633 (8.4)
Completed high school	5999 (60.4)	39 505 (62.5)	492 248 (61.4)
Completed college	3262 (32.8)	18 516 (29.3)	241 301 (30.1)
Maternal hypertension	3237 (32.6)	13 922 (22.0)	85 174 (10.6)
Breech	2971 (29.9)	5761 (9.1)	48 384 (6.0)
Fetal distress	718 (7.2)	3436 (5.4)	40 869 (5.1)
Oligohydramnios	72 (0.7)	411 (0.7)	1703 (0.2)
Polyhydramnios	17 (0.2)	102 (0.2)	532 (0.1)
Cord prolapse	701 (7.1)	6888 (10.9)	103 578 (12.9)
Rh isoimmunization	2 (0.0)	10 (0.0)	12 (0.0)
Placenta abruption	1823 (18.3)	5058 (8.0)	25 186 (3.1)
Antenatal steroids	1817 (18.3)	2892 (4.6)	13 620 (1.7)
Maternal-newborn link	7465 (75.1)	52 478 (83.1)	664 089 (82.9)
**Newborn characteristics**			
Birth weight, mean (SD), g	1028.9 (289.6)	2664.0 (409.4)	3318.7 (383.4)
Gestational age, mean (SD), wk	27.6 (2.6)	35.4 (0.8)	38.9 (1.0)
Sex			
Male	4884 (49.1)	29 476 (46.7)	393 205 (49.1)
Female	5054 (50.9)	33 684 (53.3)	407 977 (50.9)
Outborn (transferred)	1394 (14.0)	1189 (1.9)	4291 (0.5)
Congenital anomalies	1503 (15.1)	2523 (4.0)	9879 (1.2)
Key diagnosis^b^	9680 (97.4)	37 669 (59.6)	240 436 (30.0)
Major procedure	1177 (11.8)	790 (1.3)	1740 (0.2)
Death during newborn inpatient episode	1210 (12.2)	267 (0.4)	447 (0.1)
Newborn utilization			
NICU admission	9469 (95.3)	24 979 (39.5)	52 945 (6.6)
Special care days per newborn, mean (SD)	54.8 (49.8)	4.4 (11.0)	0.6 (4.6)
Length of stay, mean (SD), d	57.4 (53.8)	5.6 (12.2)	2.1 (5.5)
30-d postdischarge adverse events	1398 (14.1)	5857 (9.3)	53 381 (6.7)

Abbreviation: NICU, neonatal intensive care unit.

^a^ All cohorts restricted to birth weight of 400 g and greater and gestational age of 22 weeks or longer, excluding birth weight in less than the 3rd and greater than the 97th percentile for gestational age, limited to births within hospitals with NICU levels II to IV.

^b^ Presence of a diagnosis indicating a possible need for advanced care.

The overall NICU bed capacity was 1.02/100 LBs/year with a median across hospital-years of 0.84 (IQR, 0.57-1.30; range, 0.14-9.65). Newborns born in hospitals with higher capacity were generally more likely to have higher health risks (Table 2). Higher capacity hospitals had higher birth volumes, were more likely to be for profit, and were more likely to have a level III or IV NICU. Except for NICU admissions among VLBW newborns, NICU admission and SCD rates were higher in higher-capacity hospitals.

**Table 2.** Characteristics of Texas Medicaid Live Births and Hospitals, With Level II, III, and IV NICUs, by Median Newborn Adjusted NICU Bed Capacity, 2010 to 2014^a^

Newborn characteristics	Newborns by subcohort and hospital-adjusted NICU bed capacity, No. (%)					
	Very low birth weight (<1500 g)		Late preterm (34-36 wk)		Nonpreterm (≥37 wk)	
	Lower (295 H-Ys; 3199 LBs)	Higher (296 H-Ys; 6739 LBs)	Lower (n326 H-Ys; 27 381 LBs)	Higher (326 H-Ys; 35 779 LBs)	Lower (326 H-Ys; 374 404 LBs)	Higher (n327 H-Ys; 426 778 LBs)
Estimated neonatal mortality risk/1000 LBs, mean (SD)	1.17 (1.69)	1.25 (1.78)	0.04 (0.05)	0.04 (0.06)	0.01 (0.01)	0.01 (0.01)
Congenital anomalies	483 (15.1)	1020 (15.14)	915 (3.34)	1608 (4.49)	4309 (1.15)	5570 (1.31)
Key diagnosis	3128 (97.78)	6552 (97.23)	15 697 (57.33)	21 972 (61.41)	107 090 (28.6)	133 346 (31.24)
Major procedure	321 (10.03)	856 (12.7)	201 (0.73)	589 (1.65)	559 (0.15)	1181 (0.28)
Newborn utilization and outcomes						
NICU admission	3040 (95.03)	6429 (95.4)	9665 (35.3)	15 314 (42.8)	19 662 (5.25)	33 283 (7.8)
Special care days/newborn, mean (SD)	52 (48.88)	56 (50.12)	3.51 (9.11)	5.07 (12.29)	0.42 (4.33)	0.72 (4.89)
Length of stay, mean (SD), d	55 (53.19)	58 (54.13)	4.67 (9.67)	6.25 (13.75)	1.93 (5.17)	2.28 (5.75)
Death during newborn inpatient episode	386 (12.07)	824 (12.23)	82 (0.3)	185 (0.52)	129 (0.03)	318 (0.07)
30-d adverse events	454 (14.19)	944 (14.01)	2391 (8.73)	3466 (9.69)	23 621 (6.31)	29 760 (6.97)
Hospital characteristics						
Mean annual live births volume per hospital^a^						
Total, mean (SD)	24 (25)	52 (52)	205 (163)	241 (173)	1986 (1230)	2194 (1579)
Medicaid, mean (SD)	11 (13)	23 (23)	84 (91)	110 (104)	1148 (1157)	1305 (1317)
Teaching hospitals^b^	5 (1.69)	34 (11.49)	4 (1.23)	36 (11.04)	4 (1.23)	36 (11.01)
For profit	116 (39.32)	124 (41.89)	128 (39.26)	134 (41.1)	128 (39.26)	135 (41.28)
Nursery level						
II	126 (42.71)	30 (10.14)	149 (45.71)	46 (14.11)	149 (45.71)	47 (14.37)
III	161 (54.58)	236 (79.73)	170 (52.15)	249 (76.38)	170 (52.15)	249 (76.15)
IV	8 (2.71)	30 (10.14)	7 (2.15)	31 (9.51)	7 (2.15)	31 (9.48)

Abbreviations: H-Ys, hospital-years; LBs, live births; NICU, neonatal intensive care unit.

^a^ Less than hospital median of 0.8584 adjusted advanced care beds per 100 live births in very low birth weight; 0.8417 in late preterm; 0.8422 in nonpreterm.

^b^ Ascertained according to the presence of a neonatology fellowship.

### Adjusted Associations of NICU Capacity and Utilization

After adjustment for maternal, newborn, and hospital characteristics, NICU bed capacity was associated with utilization in LPT and NPT newborns but not VLBW newborns (Figure). Compared with the lowest capacity category (<0.50 beds/100 LBs), LPT newborns in the hospitals with highest capacity (≥2.00 beds/100 LBs) had 17% higher NICU admission (aRR, 1.17; 95% CI, 1.03-1.33). In NPT newborns, NICU admission rates were 55% higher (aRR, 1.55; 95% CI, 1.22-1.97). Numbers of SCDs were 21% and 37% higher in high bed capacity hospitals for newborns born LPT and NPT, respectively (LPT newborns: aRR, 1.21; 95% CI, 1.08-1.36; NPT newborns: aRR, 1.37; 95% CI, 1.08-1.74). Except among VLBW newborns, the tests for trend were positive (*P* < .001).

**Figure.**
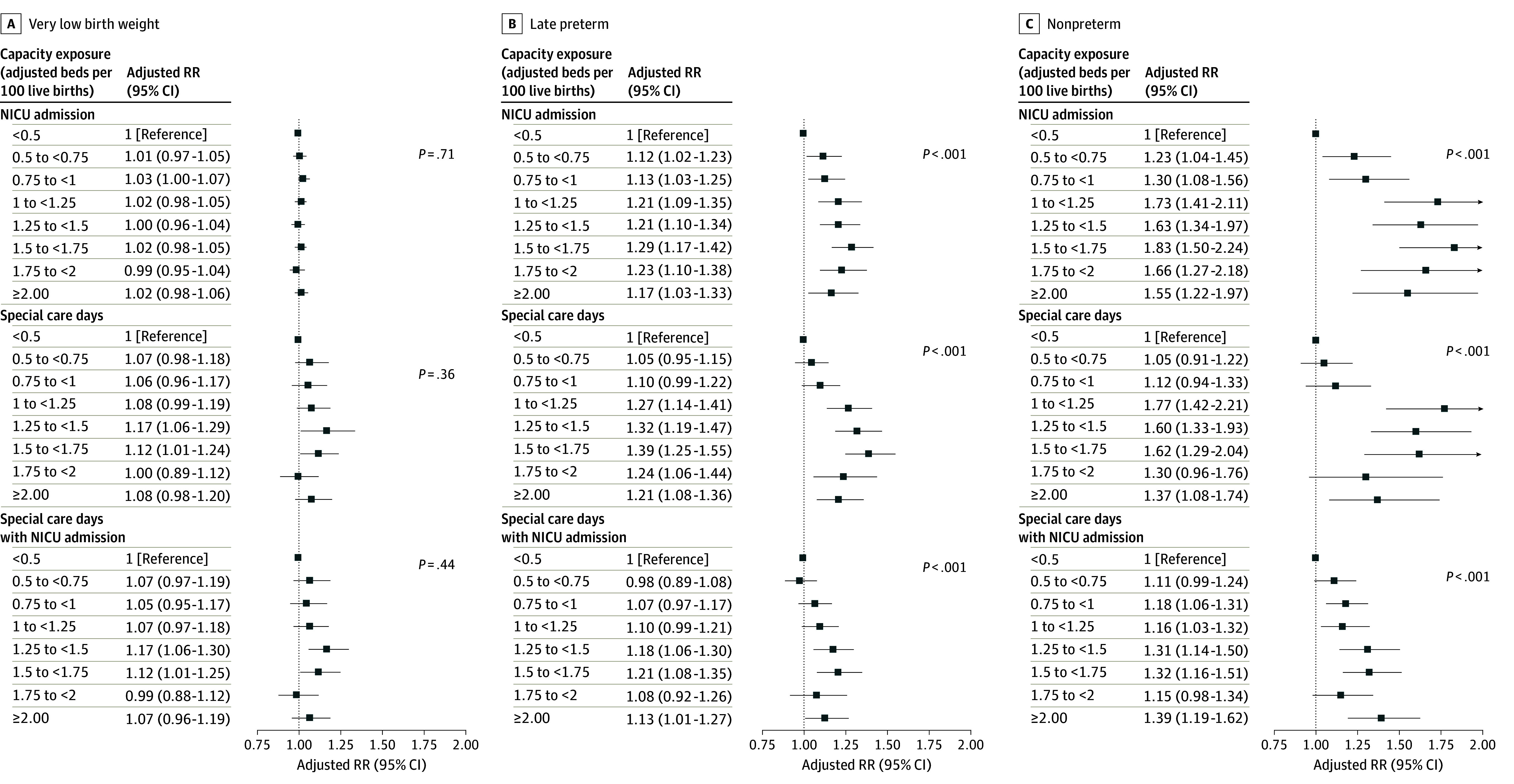
Association of Hospital-Level Neonatal Intensive Care Unit (NICU) Beds per Live Births and Inpatient Utilization, Texas Medicaid, 2010-2014 Adjusted relative risks (RRs) and 95% CIs are presented for each newborn subcohort (very low birth weight [<1500 g], late preterm [24-26 weeks’ gestation], and nonpreterm [≥37 weeks’ gestation]) for the association of hospital level NICU beds per live births with utilization. Utilization includes NICU admission, the number of special care days, and the number of special care days for newborns admitted to a NICU. Hospitals are limited to those with Medicaid-insured births and a level II to IV nursery. Relative risks were adjusted for estimated inpatient mortality categories, diagnoses, procedures, congenital anomalies; hospital covariates were volume, profit status, and presence of neonatal fellowship.

The associations of SCDs with bed capacity, conditional on NICU admission, were not statistically significant in VLBW newborns. The associations in LPT and NPT with SCDs and NICU admission were similar to the overall subcohorts.

### Stratification by Hospital Characteristics

Hospitals were stratified by birth volume, for-profit status, and teaching status (eFigures 3-5 in Supplement 1). While in 1 instance (NICU admissions for VLBW newborns in the higher volume stratum), a negative association with capacity was observed, in other strata and utilization, the association with capacity was either positive or absent.

### Adjusted Associations of NICU Capacity and Health Outcomes

NICU capacity was not associated with inpatient mortality or 30-day postdischarge composite adverse events in VLBW infants (Table 3). Compared with the lowest capacity category, in the highest category, there was, however, a moderately positive association in newborns born LPT (inpatient mortality: aRR, 1.45; 95% CI, 0.81-2.60; 30-day postdischarge events: aRR, 1.14; 95% CI, 0.92-1.43; *P* for trend < .001) and NPT (inpatient mortality: aRR, 1.80; 95% CI, 1.17-2.75; 30-day postdischarge events: aRR: 1.16; 95% CI: 0.92-1.47; *P* for trend < .001).

**Table 3.** Association of Hospital-Level Neonatal Intensive Care Unit Beds per Live Births and Newborn Adverse Events, Texas Medicaid, 2010 to 2014

Beds per 100 live births	Adjusted risk ratios (95% CI)^a^		
	Very low birth weight (<1500 g)	Late preterm (34-36 wk)	Non-preterm (≥37 wk)
**Inpatient mortality**			
<0.50	1 [Reference]	1 [Reference]	1 [Reference]
0.50 to <0.75	0.85 (0.70-1.04)	1.00 (0.61-1.66)	0.63 (0.43-0.94)
0.75 to <1.00	0.84 (0.69-1.03)	0.85 (0.50-1.44)	0.57 (0.36-0.88)
1.00 to <1.25	0.91 (0.74-1.13)	0.79 (0.43-1.46)	1.07 (0.66-1.71)
1.25 to <1.50	0.67 (0.55-0.83)	1.42 (0.86-2.34)	1.15 (0.68-1.94)
1.50 to <1.75	0.71 (0.57-0.90)	1.10 (0.64-1.88)	1.38 (0.91-2.09)
1.75 to <2.00	0.65 (0.49-0.85)	1.24 (0.70-2.20)	1.25 (0.78-2.00)
≥2.00	0.88 (0.70-1.11)	1.45 (0.81-2.60)	1.80 (1.17-2.75)
*P* for trend^b^	.95	<.001	<.001
**30-d postdischarge adverse events**			
<0.50	1 [Reference]	1 [Reference]	1 [Reference]
0.50 to <0.75	0.90 (0.72-1.12)	0.99 (0.86-1.14)	0.93 (0.82-1.04)
0.75 to <1.00	0.95 (0.75-1.20)	1.13 (0.99-1.29)	1.02 (0.92-1.14)
1.00 to <1.25	0.90 (0.71-1.15)	1.05 (0.90-1.23)	1.01 (0.88-1.18)
1.25 to <1.50	1.01 (0.81-1.25)	1.11 (0.97-1.26)	1.13 (0.99-1.29)
1.50 to <1.75	0.96 (0.75-1.23)	1.19 (1.02-1.39)	1.19 (1.01-1.40)
1.75 to <2.00	1.06 (0.79-1.42)	1.42 (1.19-1.69)	1.42 (1.15-1.75)
≥2.00	0.89 (0.69-1.16)	1.14 (0.92-1.43)	1.16 (0.92-1.47)
*P *for trend^b^	.16	<.001	<.001

^a^ Poisson generalized estimating equation models; newborn covariates were estimated inpatient mortality categories, diagnoses, procedures, congenital anomalies; hospital covariates were volume, profit status, and presence of neonatal fellowship. Inpatient mortality model for nonpreterm infants would not converge, so estimated mortality was specified as a continuous variable. Baseline rates for inpatient mortality were 11.76% (very low birth weight), 0.43% (late preterm), and 0.06% (nonpreterm). Baseline rates for 30-day postdischarge adverse events were 14.01% (very low birth weight), 9.24% (late preterm), and 6.67% (nonpreterms).

^b^ *P* value and direction of association from linear test of trend with capacity as a continuous variable. All statistically significant tests for trend indicate positive associations.

### Sensitivity Analyses

Including all birth hospitals (levels I-IV) or limiting the births to level III to IV hospitals did not generally alter study findings (eTables 1-4 in Supplement 1). The exception was an association of capacity with NICU admission for VLBW newborns when level I hospitals were included. This should be interpreted cautiously; level I NICU allocated bed capacity was very low (ie, these hospitals had no physical NICU beds); NICU admissions in infants born at level I hospitals would have required transfer to a level II to IV hospital.

## Discussion

In this population-based study of Texas Medicaid-insured newborns, we found risk-adjusted associations between hospitals’ number of NICU beds per LB and the probability of NICU admission and the number of SCDs. The strength of associations differed by subcohort. In VLBW infants, there was no association with NICU admissions—almost all newborns received NICU care—nor with SCDs. However, NICU capacity was associated with utilization in LPT and NPT newborns. There was no evidence of decreased mortality or 30-day adverse outcomes with higher capacity among VLBW newborns; in contrast, for LPT and NPT newborns, risk-adjusted inpatient mortality and 30-day adverse event rates trended higher in higher capacity hospitals.

These findings extend previous research examining the implications of variation in health care capacity and utilization. While these associations have been well-studied in adult populations,^22,23,24,25,26,27,28,29,30^ investigation in perinatal populations has been hindered by poor availability of population-based data. One known characteristic of NICU capacity is that in the past 3 decades, it has varied widely across health service regions but is unrelated to indicators of medical need.^1,31,32,33^ The supply of NICU beds does, however, appear to affect newborn utilization.

To our knowledge, the single study^32^ that examined the association between NICU capacity and utilization found a positive association between regional NICU bed supply and admissions, most strongly in lower risk newborn groups. However, regional supply of NICU beds is an average across many hospitals, failing to account for the heterogeneity of capacity exposure. Others have investigated the association indirectly. Haberland et al^34^ reported that growth in California midlevel units and bed supply was associated with shifts of VLBW newborns to these lower care–level units. Profit and colleagues^35^ found in moderately preterm newborns from 2 states that during days with a higher NICU census, the likelihood of discharge was higher, but without any observed untoward outcomes for parents or newborns. Freedman^36^ reported that within-hospital monthly variation in unused NICU beds in California and New York was associated with higher NICU admissions, particularly for newborn groups of lower average risk. The current study extends the inference of these previous papers with hospital-level capacity exposure to a large, diverse, and vulnerable newborn population.

In nonpediatric health care research, the association of capacity with utilization is accompanied by weak or absent population-level benefits, suggesting overuse.^23,25,27,28,29,37^ The exception to this generality is found in regions with extremely low capacity, in services such as primary care, but only a small fraction of the US population resides in underresourced health care markets. One recent neonatal study^38^ reported that short-term outcomes were not worse in hospitals with lower levels of adjusted NICU utilization. The current study extends the utilization-outcome relationship to NICU hospital capacity in a multilevel model and failed to detect adverse consequences of lower capacity at a population level.

The finding of worse health outcomes for LPT and NPT newborns in hospitals with higher capacity was unexpected. This association may be explained by residual confounding. Another possibility is that hospital medical care quality and outcomes in lower risk newborns varies similarly to the variation well documented in newborns born with a weight of less than 1500 g.^8^ If so, the factors associated with these differences are poorly understood, as evidenced in an article by Salazar et al.^21^ They reported heterogeneity in unit care quality of medium preterm and LPT newborns, with worse quality associated with care in higher level NICUs.^21^

There is a growing body of evidence that a high proportion of NICU-admitted newborns have relatively low-severity illness^7,9,41^ and that there are opportunities to reduce admissions and lengths of NICU stays.^5,38,42^ In the 20 years since Goodman et al^22,31,43^ described regional variation in NICU capacity and an absent association of NICU regional bed supply with neonatal mortality, there has been robust further growth in the number of NICU beds. Capacity is associated with higher NICU utilization in the lower-risk newborn groups, the newborns who have experienced the strongest secular increase in NICU admission rates. In absolute numbers, LPT and NPT newborns are the most affected groups, and their care has received less research or clinical improvement effort than for newborns born VLBW.^44^ This should be of concern for 3 reasons. The first is that it is hard to imagine a scenario where capacity location unrelated to newborn needs would not lead to lower quality, with higher costs to society and families. Second, this study and others have shown that higher capacity levels, whether measured at a regional or hospital level, are associated with higher NICU use. There are some newborns who benefit from this greater availability, but the strongest effects of capacity are found in the lowest-need newborn groups, where the possibility of overuse is highest. And third, to date, across the observed variation in risk-adjusted NICU utilization, higher rates are not associated with population benefits. This suggests that either the wrong newborns are receiving NICU care or that many infants could be cared for in non-NICU hospital settings or discharged earlier without harm. Even in the absence of definitive evidence of overuse, some health systems have successfully reduced the use of NICUs for lower-risk groups.^5,12^

Our understanding of the causes and consequences of variation in NICU capacity and utilization remains incomplete. However, taken with previous research, our study suggests that there may be overcapacity of NICUs in some health care markets and overuse of NICU care in some newborn populations. Given the high costs associated with training neonatal clinicians, adding NICU beds, paying for the associated NICU utilization, and the unintended clinical consequences of NICU care, further investigation into the benefits of additional NICU capacity expansion is warranted.

### Limitations

This study has limitations. Our findings may not be generalizable to newborns in other states or not insured by Medicaid. Texas births, however, exceed 10% of US births, with most insured by Medicaid. An advantage of this dataset is the inclusion of both maternal and infant facility and professional encounters for all Medicaid births linked to birth and death vital records. Nevertheless, this dataset is difficult to replicate at larger population scales and may have lower data quality than that found in clinical registries.

The exposure measure, allocated NICU beds per LB, accounts for newborn transfers by assigning beds occupied by transfers to the birth hospital. It assumes, however, that a local bed has similar associations with utilization as the distant beds.

As an observational study, we cannot rule out residual confounding. Specifically, the models estimating newborn mortality had high discrimination power for the VLBW and LPT cohorts, but less so for the NPT cohort. Methods for newborn risk adjustment are not well developed in lower risk groups, where mortality is less common and available covariates for other outcomes, such as readmission, are endogenous to medical care. Maternal and newborn morbidity has changed over the past 2 decades, with sharp increases in maternal obesity and opioid-exposed mothers; illness from these factors is more difficult to measure in higher gestational age cohorts.^32^ In the current study, capacity was measured at the hospital level; as is evident from the measured hospital characteristics, higher capacity hospitals tended to care for newborns with higher illness acuity. This fact may explain higher rates of mortality in hospitals with higher bed supply even after risk adjustment. We attempted to reduce this bias with covariates that accounted for the presence of diagnoses associated with the need for NICU care; however, higher utilization, including NICU admission, all else held equal, is likely to lead to more diagnoses. Importantly, previous studies in adult populations using administrative data for risk adjustment have found a bias that results in the underestimation of actual associations, (ie, an overadjustment).^39,40^

Furthermore, our outcome measures were limited to observing 3 events up to 30 days after discharge. Other events indicating potential benefits and harms are not captured; some of these outcomes, such as early and later life neurodevelopment, are important areas for future research.

## Conclusions

In this cohort study of Texas Medicaid-insured newborns, greater hospital NICU bed supply was associated with increased NICU utilization in newborns born LPT and NPT. Higher capacity was not associated with lower risks of adverse events.

## Notes

Supplement 1. **eAppendix.** Supplementary Methods **eFigure 1.** Texas Medicaid Newborn Study Cohort, 2010-2014 **eFigure 2.** NICU Bed Count Allocation Method **eFigure 3.** Associations of Hospital Allocated Neonatal Intensive Care Beds per Live Birth With Utilization, Stratified by Median Hospital Total Live Births, Texas Medicaid-Insured Newborns, 2010-2014 **eFigure 4.** Associations of Hospital Allocated Neonatal Intensive Care Beds per Live Birth With Utilization, Stratified by Hospital Profit Status, Texas Medicaid-Insured Newborns, 2010-2014 **eFigure 5.** Associations of Hospital Allocated Neonatal Intensive Care Beds per Live Birth With Utilization, Stratified by Presence of Hospital Neonatal Fellowship, Texas Medicaid-Insured Newborns, 2010-2014 **eTable 1.** Association of Hospital-Level Advanced Care Beds per Live Birth and Inpatient Utilization With All Hospital Nursery Levels, Texas Medicaid, 2010-2014 **eTable 2.** Association of Hospital-Level Advanced Care Beds per Live Birth and Inpatient Utilization With Hospital Nursery Levels III and IV, Texas Medicaid, 2010-2014 **eTable 3.** Association of NICU Beds per Live Births and Newborn Adverse Events With All Hospital Nursery Levels, Texas Medicaid, 2010-2014 **eTable 4.** Association of NICU Beds per Live Births and Newborn Adverse Events With Hospital Nursery Levels III and IV, Texas Medicaid, 2010-2014

Supplement 2.
Nonauthor Collaborators

Supplement 3.
Data Sharing Statement
