# Supplementary material for: Hospital-Level NICU Capacity, Utilization, and 30-Day Outcomes in Texas
Source: JAMA Netw Open. 2024 Feb 14;7(2):e2355982. doi: 10.1001/jamanetworkopen.2023.55982 (PMC10867701; doi:10.1001/jamanetworkopen.2023.55982)
Supplement: Supplement 1. — eAppendix. Supplementary Methods eFigure 1. Texas Medicaid Newborn Study Cohort, 2010-2014 eFigure 2. NICU Bed Count Allocation Method eFigure 3. Associations of Hospital Allocated Neonatal Intensive Care Beds per Live Birth With Utilization, Stratified by Median Hospital Total Live Births, Texas Medicaid-Insured Newborns, 2010-2014 eFigure 4. Associations of Hospital Allocated Neonatal Intensive Care Beds per Live Birth With Utilization, Stratified by Hospital Profit Status, Texas Medicaid-Insured Newborns, 2010-2014 eFigure 5. Associations of Hospital Allocated Neonatal Intensive Care Beds per Live Birth With Utilization, Stratified by Presence of Hospital Neonatal Fellowship, Texas Medicaid-Insured Newborns, 2010-2014 eTable 1. Association of Hospital-Level Advanced Care Beds per Live Birth and Inpatient Utilization With All Hospital Nursery Levels, Texas Medicaid, 2010-2014 eTable 2. Association of Hospital-Level Advanced Care Beds per Live Birth and Inpatient Utilization With Hospital Nursery Levels III and IV, Texas Medicaid, 2010-2014 eTable 3. Association of NICU Beds per Live Births and Newborn Adverse Events With All Hospital Nursery Levels, Texas Medicaid, 2010-2014 eTable 4. Association of NICU Beds per Live Births and Newborn Adverse Events With Hospital Nursery Levels III and IV, Texas Medicaid, 2010-2014 [file jamanetwopen-e2355982-s001.pdf]

Supplemental Online Content

Goodman DC, Stuchlik P, Ganduglia-Cazaban C, et al; Texas Neonatal Care Research Collaborative. Hospital-level NICU capacity, utilization, and 30-day outcomes in Texas. *JAMA Netw Open.* 2024;7(2):e2355982. doi:10.1001/jamanetworkopen.2023.55982

eAppendix. Supplementary Methods

eFigure 1. Texas Medicaid Newborn Study Cohort, 2010-2014

eFigure 2. NICU Bed Count Allocation Method

eFigure 3. Associations of Hospital Allocated Neonatal Intensive Care Beds per Live Birth With Utilization, Stratified by Median Hospital Total Live Births, Texas Medicaid-Insured Newborns, 2010-2014

eFigure 4. Associations of Hospital Allocated Neonatal Intensive Care Beds per Live Birth With Utilization, Stratified by Hospital Profit Status, Texas Medicaid-Insured Newborns, 2010-2014

eFigure 5. Associations of Hospital Allocated Neonatal Intensive Care Beds per Live Birth With Utilization, Stratified by Presence of Hospital Neonatal Fellowship, Texas Medicaid-Insured Newborns, 2010-2014

eTable 1. Association of Hospital-Level Advanced Care Beds per Live Birth and Inpatient Utilization With All Hospital Nursery Levels, Texas Medicaid, 2010-2014

eTable 2. Association of Hospital-Level Advanced Care Beds per Live Birth and Inpatient Utilization With Hospital Nursery Levels III and IV, Texas Medicaid, 2010-2014

eTable 3. Association of NICU Beds per Live Births and Newborn Adverse Events With All Hospital Nursery Levels, Texas Medicaid, 2010-2014

eTable 4. Association of NICU Beds per Live Births and Newborn Adverse Events With Hospital Nursery Levels III and IV, Texas Medicaid, 2010-2014

This supplemental material has been provided by the authors to give readers additional information about their work.

© 2024 Goodman DC et al. *JAMA Network Open.*

eAppendix. Supplementary Methods

Texas Medicaid Neonatal Intensive Care Unit Project
Risk Adjustment Models

Texas Medicaid Newborn Cohort.

The Texas Medicaid Newborn Cohort is a retrospective cohort of over a million TX newborns insured by Medicaid/CHIP, born 1/1/2010 to 12/31/2014, characterized through linkage of Medicaid newborn enrollment files to natality records (92.8% linkage), mortality records for the first year of life (96% linkage), and maternal Medicaid enrollment records (88% linkage). See eFigure 1 (at the end of this document) Maternal linkage rates varied across regions (79%–96%) but did not correlate with utilization rates (Spearman rho ≥ 0.1). Records were further linked to maternal/newborn facility (i.e., hospital) and professional claims/encounters. Claims end on 12/31/15 for 2014 births. TX Medicaid-managed care plans report all encounter records to the State.

Risk Adjustment

In calculating utilization across regions and hospitals, rates were adjusted through two methods: restriction and multi-variable regression. Restriction assigns newborns to cohorts of similar average risk, such as very low birth weight or late pre-term newborns. Further adjustment for differences within the restricted cohorts used multivariable modeling.

The development of the risk adjustment models builds on the study conceptual framework (Methods appendix Figure 1). The outcomes of interest in this study are post discharge events (i.e. processes of care and mortality), but the framework also applies to inpatient newborn utilization.

The ideal covariates are those maternal and newborn characteristics *present before or at the time of birth that are unaffected by newborn care*. Diagnoses subsequent to birth (e.g., respiratory distress syndrome) also indicate health status, but it should be noted that these can be affected by medical care and different thresholds of diagnostic labeling; therefore, the inclusion of clinical diagnoses and medical care events post-birth tend to over adjust for health status differences across hospitals.

© 2024 Goodman DC et al. *JAMA Network Open.*

Conceptual Framework. Methods appendix figure 1. Study conceptual framework. Primary exposures and outcomes are listed across the trajectory of pregnant women → inpatient newborns → post-discharge hospitalization. Note that some outcome variables serve as exposure variables in the temporal periods under study.

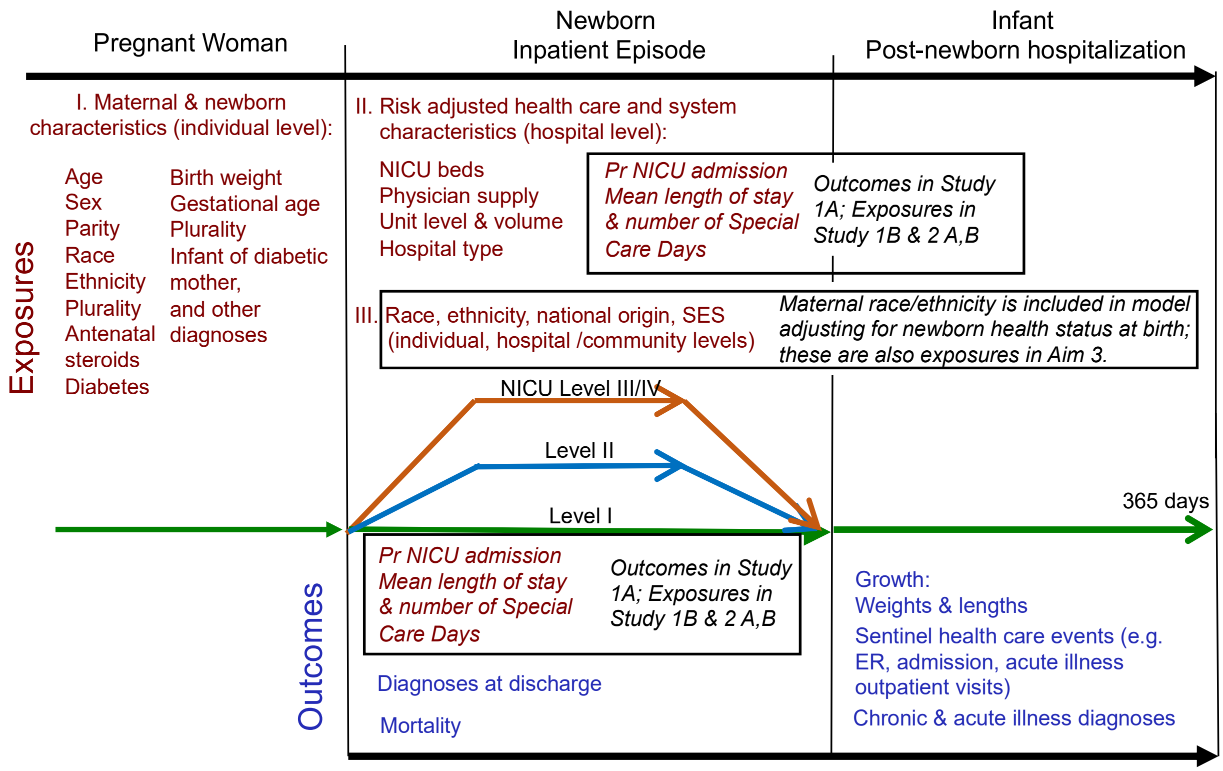

There is no single measure of health status at birth that can be used for risk adjustment by itself, even if it were feasible to collect additional primary data for each newborn. Instead, in the first step, the model estimates the probability of 27-day mortality at the newborn level using maternal and newborn characteristics included in the natality, enrollment, and claims/events files. The purpose of the model is not to explain the causes of neonatal mortality, but to derive the best parsimonious prediction of mortality, while maintaining clinical face validity and incorporating predictors demonstrated in previous studies. This first model, specific to each cohort, was used to calculate a risk score (i.e. predicted probability of 27-day mortality) for each newborn within their respective cohort.

Deaths were ascertained from the linked mortality file and from any additional occurrences reported on a hospitalization claim/encounter. Death was chosen as the outcome for model development because it is measurable, relatively common in this population, and important.

Most deaths from perinatal causes occur within the first few days of life (Appendix Figure 2), but deaths from illness related to prematurity or congenital anomalies can occur later, so sensitivity testing was conducted with models using 60 and 90-day mortality.

© 2024 Goodman DC et al. *JAMA Network Open.*

Figure 2 – Probability of mortality in first 90 days of life (Source 2010 National Birth-Linked Death File, CDC).

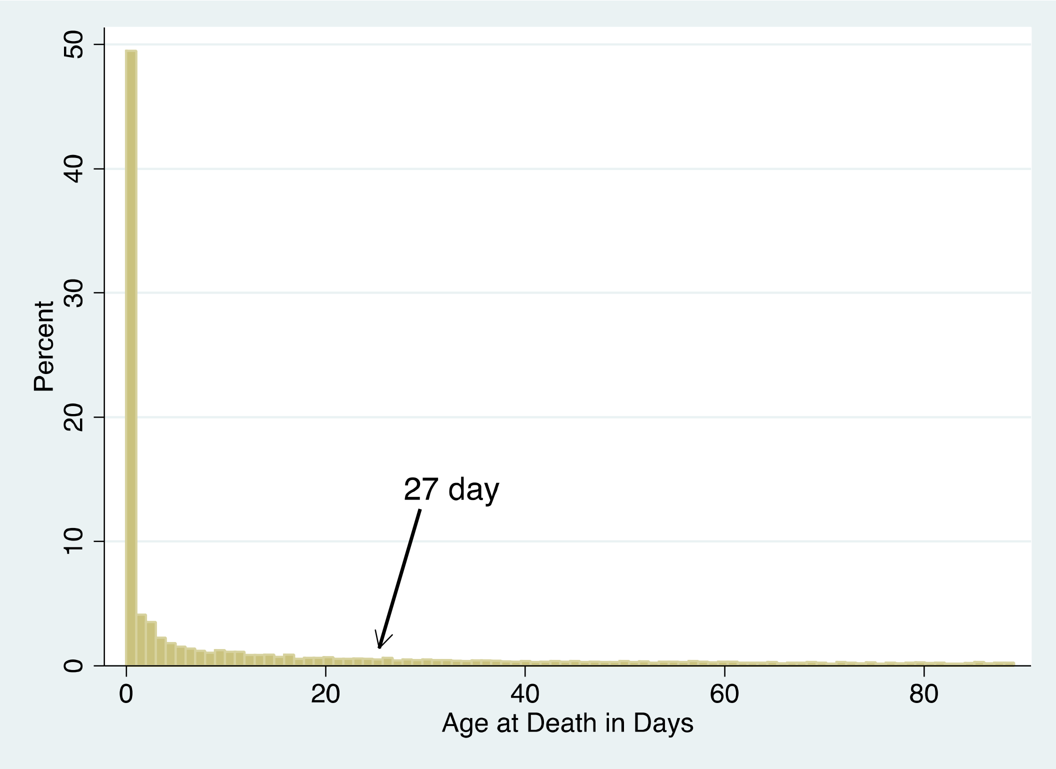

Candidate risk factors were derived through a systematic literature search of papers that developed predictive or explanatory models of neonatal mortality. (See references at the end of Appendix) From these, we identified three papers which modeled neonatal mortality using data from natality and utilization files:

Chung, J. H., C. S. Phibbs, W. J. Boscardin, G. F. Kominski, A. N. Ortega, and J. Needleman. "The Effect of Neonatal Intensive Care Level and Hospital Volume on Mortality of Very Low Birth Weight Infants." *Medical Care* 48, no. 7 (2010): 635–44.

Phibbs, C. S., L. C. Baker, A. B. Caughey, B. Danielsen, S. K. Schmitt, and R. H. Phibbs. "Level and Volume of Neonatal Intensive Care and Mortality in Very-Low-Birth-Weight Infants." *N Engl J Med* 356, no. 21 (2007): 2165–75.

Goodman, D. C., E. S. Fisher, G. A. Little, T. A. Stukel, C. H. Chang, and K. S. Schoendorf. "The Relation between the Availability of Neonatal Intensive Care and Neonatal Mortality." *N Engl J Med* 346, no. 20 (May 16, 2002): 1538–44.

Covariates were identified, and comparable variables were generated for each newborn in the overall birth cohort (live births ≥400 grams). Model building is discussed in detail for Cohort 1

© 2024 Goodman DC et al. *JAMA Network Open.*

(all live births >400 grams) and model summaries presented for the specific cohorts used in this paper.

Cohort 1 (live births ≥400 grams)

<u>Univariate Analysis</u>

Tabulations of study variables are presented in two tables. Appendix Table 1 reports variables from the natality file. Appendix Table 2 includes variables from facility and professional claims diagnoses (claims codes are found in Appendix Table 3). If there was no linkage with the maternal Medicaid record, and an absence of the factor on the natality and infant Medicaid file, the variable was coded as unknown. An absent linkage was included as a model covariate. Appendix Table 4 are congenital anomalies associated with mortality adapted from Chung, et. al. paper (See page 3)

© 2024 Goodman DC et al. *JAMA Network Open.*

Appendix Table1. Candidate variables from Natality File.

BIRTH WEIGHT	Frequency	Percent	Cummulative Frequency	Cumuluative Percent
400-499g	756	0.07%	756	0.1%
500-599g	1164	0.10%	1920	0.2%
600-699g	1231	0.11%	3151	0.3%
700-799g	1449	0.13%	4600	0.4%
800-899g	1297	0.11%	5897	0.5%
900-999g	1627	0.14%	7524	0.7%
1000-1099g	1404	0.12%	8928	0.8%
1100-1199g	1756	0.15%	10684	0.9%
1200-1299g	1582	0.14%	12266	1.1%
1300-1399g	2092	0.18%	14358	1.3%
1400-1499g	2039	0.18%	16397	1.4%
1500-1599g	2934	0.26%	19331	1.7%
1600-1699g	2700	0.24%	22031	1.9%
1700-1799g	3888	0.34%	25919	2.3%
1800-1899g	4968	0.44%	30887	2.7%
1900-1999g	5011	0.44%	35898	3.2%
2000-2099g	7775	0.69%	43673	3.8%
2100-2199g	7924	0.70%	51597	4.5%
2200-2299g	12911	1.14%	64508	5.7%
2300-2399g	14072	1.24%	78580	6.9%
2400-2499g	22297	1.97%	100877	8.9%
2500-2599g	24204	2.13%	125081	11.0%
2600-2699g	38976	3.43%	164057	14.5%
2700-2799g	42901	3.78%	206958	18.2%
2800-2899g	66492	5.86%	273450	24.1%
2900-2999g	66674	5.88%	340124	30.0%
3000-3099g	93720	8.26%	433844	38.2%
3100-3199g	84046	7.41%	517890	45.6%
3200-3299g	106778	9.41%	624668	55.1%
3300-3399g	86486	7.62%	711154	62.7%
3400-3499g	97267	8.57%	808421	71.2%
3500-3599g	68712	6.06%	877133	77.3%
3600-3699g	70589	6.22%	947722	83.5%
3700-3799g	55378	4.88%	1003100	88.4%
3800-3899g	36101	3.18%	1039201	91.6%
3900-3999g	31604	2.79%	1070805	94.4%
4000-4099g	18672	1.65%	1089477	96.0%
4100-4199g	16011	1.41%	1105488	97.4%
4200-4299g	9173	0.81%	1114661	98.2%
4300-4399g	7355	0.65%	1122016	98.9%
4400-4499g	4062	0.36%	1126078	99.2%
4500-4599g	3269	0.29%	1129347	99.5%
4600-4699g	1691	0.15%	1131038	99.7%
4700-4799g	1345	0.12%	1132383	99.8%
4800-4899g	755	0.07%	1133138	99.9%
4900-4999g	566	0.05%	1133704	99.9%
5000-5099g	310	0.03%	1134014	99.9%
5100-5199g	263	0.02%	1134277	100.0%
5200-5299g	109	0.01%	1134386	100.0%
5300-5399g	117	0.01%	1134503	100.0%
5400-5499g	68	0.01%	1134571	100.0%
5500-5599g	54	0.00%	1134625	100.0%
5600-5699g	37	0.00%	1134662	100.0%
5700-5799g	25	0.00%	1134687	100.0%
5800-5899g	13	0.00%	1134700	100.0%
Unknown	-99999		-99999	-99999

© 2024 Goodman DC et al. *JAMA Network Open.*

RACE	Frequency	Percent	Cumulative Frequency	Cumulative Percent
WHITE	773495	68.17	773495	68.17
BLACK	164732	14.52	938227	82.68
ASIAN	25743	2.27	963970	84.95
NATIVE AMERICAN	3673	0.32	967643	85.28
OTHER	167060	14.72	1134703	100.00

HISPANIC	Frequency	Percent	Cumulative Frequency	Cumulative Percent
NO	431475	38.03	431475	38.03
YES	703228	61.97	1134703	100.00

EDUCATION	Frequency	Percent	Cumulative Frequency	Cumulative Percent
8 years or fewer	91569	8.07	91569	8.07
Some highschool	295992	26.09	387561	34.16
Completed highschool	401431	35.38	788992	69.53
College	344811	30.39	1133803	99.92
Unknown	900	0.08	1134703	100.00

YEAR	Frequency	Percent	Cumulative Frequency	Cumulative Percent
2010	215172	18.96	215172	18.96
2011	223342	19.68	438514	38.65
2012	230256	20.29	668770	58.94
2013	231463	20.40	900233	79.34
2014	234470	20.66	1134703	100.00

SEX	Frequency	Percent	Cumulative Frequency	Cumulative Percent
Female	554363	48.86	554363	48.86
Male	580325	51.14	1134688	100.00
Unknown	15	0.00	1134703	100.00

GES_AGE	Frequency	Percent	Cumulative Frequency	Cumulative Percent
<24 weeks	1702	0.15	1702	0.15
24-25 weeks	2656	0.23	4358	0.38
26-27 weeks	3342	0.29	7700	0.68
28-29 weeks	4465	0.39	12165	1.07
30-31 weeks	7324	0.65	19489	1.72
32-33 weeks	15075	1.33	34564	3.05
34-35 weeks	40847	3.60	75411	6.65
36-37 weeks	162440	14.32	237851	20.96
38-39 weeks	648885	57.19	886736	78.15
40-41 weeks	241578	21.29	1128314	99.44
42-43 weeks	5391	0.48	1133705	99.91
>42 weeks	638	0.06	1134343	99.97
Unknown	360	0.03	1134703	100.00

MULT_BIRTH	Frequency	Percent	Cumulative Frequency	Cumulative Percent
NO	1104979	97.38	1104979	97.38
YES	29724	2.62	1134703	100.00

SMOKING	Frequency	Percent	Cumulative Frequency	Cumulative Percent
NO	1038787	91.55	1038787	91.55
YES	95916	8.45	1134703	100.00

© 2024 Goodman DC et al. *JAMA Network Open.*

Appendix Table 2. Candidate variables from Medicaid files.

	NO<br>N<br>%	YES<br>N<br>%	UNKNOWN<br>N<br>%
**Alcohol Use**	936,353	672	197,678
	82.52%	0.06%	17.42%
**Diabetes**	808,776	142,617	183,310
	71.28%	12.57%	16.15%
**RH Isoimmunization**	936,985	45	197,673
	82.58%	0.00%	17.42%
**Breech Birth**	859,143	84,716	190,844
	75.72%	7.47%	16.82%
**Incompetent Cervix**	926,736	10,302	197,665
	81.67%	0.91%	17.42%
**Chorioamnionitis**	908,479	30,291	195,933
	80.06%	2.67%	17.27%
**Cord Prolapse**	797,386	139,739	197,578
	70.27%	12.32%	17.41%
**Drug Use**	905,199	31,993	197,511
	79.77%	2.82%	17.41%
**Fetal Distress**	882,663	57,318	194,722
	77.79%	5.05%	17.16%
**nteruterine Growth Restriction**	806,232	133,078	195,393
	71.05%	11.73%	17.22%
**Hematologic**	927,993	10,825	195,885
	81.78%	0.95%	17.26%
**Hypertension**	800,920	138,944	194,839
	70.58%	12.24%	17.17%
**Infection**	707,960	229,144	197,599
	62.39%	20.19%	17.41%
**Large for Gestational Age**	760,418	200,351	173,934
	67.01%	17.66%	15.33%
**Oligohydramnios**	934,481	2,980	197,242
	82.35%	0.26%	17.38%
**Placenta Abruption**	892,237	45,176	197,290
	78.63%	3.98%	17.39%
**Placenta Previa**	909,471	27,555	197,677
	80.15%	2.43%	17.42%
**Polyhydraminos**	936,158	990	197,555
	82.50%	0.09%	17.41%
**Preeclampsia**	810,492	136,110	188,101
	71.43%	12.00%	16.58%
**Preterm Labor**	719,702	217,406	197,595
	63.43%	19.16%	17.41%
**Prior Preterm Birth**	895,128	45,698	193,877
	78.89%	4.03%	17.09%
**Renal Disease**	924,923	12,103	197,677
	81.51%	1.07%	17.42%
**Maternal Steroids**	913,956	27,490	193,257
	80.55%	2.42%	17.03%
**Congenital Anomalies**	1,080,204	54,499	0
	95.20%	4.80%	0.00%

© 2024 Goodman DC et al. *JAMA Network Open.*

Appendix Table 3. Medicaid file variables definitions (ICD-9-CM) for mortality models.

**Alcohol Use**	303.9	For all codes, include any 4th or 5th
	305.0	digit codes, unless 4th or 5th digit cod
**Diabetes Mellitus**		are specified.
	648.0	
	648.8	
	v58.67	
	775.0	
**Drug Use**		
	304.9	
	305.2	
	305.3	
	305.6	
	305.9	
	648.3	
**Hypertension**		
	401	
	402	
	404	
	405	
	642	
**Incompetent Cervix**		
	654.5	
	761.0	
**Infection**		
	646.6	
	647	
**Placenta Abruption**		
	641.2	
	656.7	
	762.1	
	762.2	
**Preeclampsia**		
	642.3	
	642.4	
	642.5	
	642.6	
	642.7	
	646.2	
**Preterm Labor**		
	644.0	
	644.2	
**Prior Preterm Birth**		
	v23.41	
**Placenta Previa**		
	641.0	
**Renal Disease**		
	646.20	
	646.21	
	646.23	
**Hematologic**		
	773.0	
	773.2	
**Intrauterine Growth Restriction**		
	656.5	
	764.1	
	764.2	
	764.9	
**Large for gestational age**	656.6	
	766	
	766.1	
**Oligohydramnios**	761.2	
**Polyhydramnios**		
	761.3	
**RH Isoimmunization**		
	773.3	
**Breech**		
	652.2	
**Chorioamnionitis**		
	658.40	
	658.41	
	658.43	
	762.7	
Cord Prolapse	663.0	
	663.1	
	663.3	
	762.4	
Fetal distress	656.8	
	768.2	
	768.3	
	768.4	
	768.5	
	768.6	
	768.9	
maternal steroids	9923	ICD-9 procedure code - facility claim
	J1100	dexamethasone phosphate injection
	J0702	betamethasone injection

Appendix Table 4. Congenital anomalies associated with mortality in published studies.

Category	Code	Name
Code each newborn with presence or absence of any anomaly		
**Abdominal Wall Defect**		
	756.79	Other congenital anomalies of abdominal wall
**Tracheoesophageal Fistula**		
	750.3	Tracheoesophageal fistula, esophageal atresia and stenosis
	750.4	Other specified anomalies of esophagus
**Small bowel or upper GI anomalies**		
	560.2	Volvulus
	750.5	Congenital hypertrophic pyloric stenosis
	750.7	Other specified anomalies of stomach
	750.8	Other specified anomalies of upper alimentary tract
	750.9	Unspecified anomaly of upper alimentary tract
	751.1	Atresia and stenosis of small intestine
	751.4	Anomalies of intestinal fixation
	751.5	Other anomalies of intestine
	751.8	Other specified anomalies of digestive system
	751.9	Unspecified anomaly of digestive system
**Meckel's Syndrome**		
	751.0	Meckel's Diverticulum
**Large bowel**		
	751.2	Atresia and stenosis of large intestine, rectum, and anal cana
	751.3	Hirschsprung's disease and other congenital functional disorders of colon
	751.61	Biliary atresia
	751.69	Other anomalies of gallbladder, bile ducts, and liver
	751.7	Anomalies of pancreas
	771.1	Congenital cytomegalovirus infection
**Genitourinary**		
	753.0	Renal agenesis and dysgenesis
	753.10	Cystic kidney disease, unspecified
	753.12	Polycystic kidney, unspecified type
	753.14	Polycystic kidney, autosomal recessive
	753.15	Renal dysplasia
	753.19	Other specified Kidney disease
	753.3	Other specified anomalies of kidney
	753.4	Other specified anomalies of ureter
**Obstructions**		
	753.20	Unspecified obstructive defect of renal pelvis and ureter
	753.21	Congenital obstruction of ureteropelvic junction
	753.22	Congenital obstruction of ureterovesical junction
	753.23	Congenital ureterocelcle
	753.6	Atresia and stenosis of urethra and bladder neck
	753.7	Anomalies of urachus
	753.8	Other specified anomalies of bladder and urethrea
	753.9	Unspecified anomaly of urinary system
	756.71	Prune Belly Syndrome
**Spina bifida**		
	741.00	Spina bifida with hydrocephalus, unspecified region
	741.01	Spina bifida with hydrocephalus, cervical region
	741.02	Spina bifida with hydrocephalus, dorsal (thoracic) region
	741.03	Spina bifida with hydrocephalus, lumbar region
	741.90	Spina bifida without mention hydrocephalus, unspecified regi
	741.91	Spina bifida without mention hydrocephalus, cervical region
	741.92	Spina bifida without mention hydrocephalus, dorsal (thoracic) region
	741.93	Spina bifida without mention hydrocephalus, lumbar region
	742.0	Encephalocele
	742.59	Other
**Brain**		
	742.1	Microcephalus
	742.2	Reduction deformaties of brain
	742.3	Congenital hydrocephalus
	742.4	Other specified anomalies of brain
	742.8	Other specified anomalies of nervous system
	742.9	Unspecified anomaly of brain, spinal cord, and nervous syste
**Congenital Diaphragmatic Hernia**		
	519.4	Disorders of Diaphram
	553.3	Diaphragmatic hernia
	750.6	Congenital hiatus hernia
	756.6	Anomalies of diaphragm
**Airway**		
	748.3	Other anomalies of larynx, trachea, and bronchus
	748.9	Unspecified anomaly of respiratory system
**Cyst**		
	748.4	Congenital cystic lung
	748.60	Anomaly of lung, unspecified
	748.69	Other
	748.8	Other specified anomalies of respiratory system

© 2024 Goodman DC et al. *JAMA Network Open.*

**Aortic Valve**		
	424.1	Aortic valve disorders
	746.3	Congenital stenosis of aoirtic valve
	746.4	Congenital insufficiency of aortic valve
**Aortic Arch**		
	747.10	Coarctation of aorta (preductal) (postductal)
	746.81	Subaortic stenosis
	747.11	Interruption of aortic arch
	747.21	Anomalies of aortic arch
	747.22	Atresia and stenosis of aorta
	747.29	Other
**Hypoplastic Left Heart Syndrome**		
	746.7	Hypoplastic left heart syndrome
**Endocardial fibroelastosis**		
	425.3	Endocardial fibroelastosis
**Mitral valve**		
Stenosis		
	746.5	Congenital mitral stenosis
Other Mitral		
	424.0	Mitral Valve disorders
	746.6	Congenital mitral insufficiency
	746.84	Obstructive anomalies of heart, NEC
**Transpositions**		
	745.10	Complete transposition of great vessels
	745.12	Corrected transposition of great vessels
	745.19	Other
**Coronary/Myocardial**		
	425.1	Hypertrophic obstructive cardiomyopathy
	746.85	Coronary artery anomaly
**Common Right Ventricle**		
	745.0	Common truncus
	745.3	Common ventricle
**Pulmonary vavle- tricuspid**		
	745.2	Tetrology of Fallot
	746.01	Atresia, congenital
	746.09	Other congenital insufficiency of pulmonary valve
	746.1	Tricuspid atresia and stenosis, congenital
	746.2	Ebstein's anomaly
	746.83	Infundibular pulmonic stenosis
**Cushion**		
	745.60	Endocardial cushion defect, unspecified type
	745.61	Ostium primum defect
	745.69	Other
**Pulmonary veins**		
	746.82	Cor triatriatum
	747.41	Total anomalous pulmonary venous
	747.42	Partial anomalous pulmonary venous connection
**Great Vein**		
	747.40	Anomaly of great veins, unspecified
	747.49	Other anomalies of great veins
**Skeletal**		
	756.50	Osteodystrophy, unspecified
	756.51	Osteogenesis imperfecta
	756.55	Chondroectodermal dysplasia
	756.59	Other
**Chromosomal Syndromes**		
	758.3	Autosomal deletion syndromes
	758.5	Other conditions due to autosomal anomalies
	758.89	Other
	758.9	Conditions due to anomaly of specified chromosome
	759.4	Conjoined twins
	759.7	Multiple congenital anomalies
	759.89	Other
	759.9	Congenital anomaly, unspecified
**Other**		
Non-Immune hydrops		
	778.0	Hydrops getalis not due to isoimmunization
Harmartoses		
	759.6	Other hamartoses, NEC
Congenital anemia		
	776.5	Congenital anemia

© 2024 Goodman DC et al. *JAMA Network Open.*

From these tabulations, it was decided to delete newborns with the unknown characteristics listed in Appendix Table 5. Unknown characteristics of higher counts were either preserved as a variable value or combined with a known category.

Appendix Table 5. Counts of newborns deleted from cohorts for unknown characteristics. Note that the three cohorts used in this paper exclude multiple births.

Cohort	Total TX Newborns	Unknown Sex	Unknown Education	Unknown Gestational Age	Total Newborns in Model
Overall	1,134,703	15	900	347	1,133,441
<1500 g	12,902	2	52	22	12,826
34-36 wk	78,093	3	77	347	77,666
≤ 37 wk	999,334	11	718	334	998,271

© 2024 Goodman DC et al. *JAMA Network Open.*

Bivariate Analyses

Each set of variables was then specified in separate logistic models (Appendix Table 6).

Appendix Table 6. Bivariate models with 27-day mortality as dependent variable.

**Variable**	27 day death		Risk	Odds ratio	P-value	C statistic
	no	yes				
**Sex**						0.501
Female	552685	1678	0.30%	0.851	<.0001	
Male	578263	2062	0.36%	1.000	---	
Unknown	9.000	6.000	40%	187.449	<.0001	
**Race**						0.54
Asian	25680	63	0.24%	0.812	0.1025	
Black	163890	842	0.51%	1.700	<.0001	
Native American	3665	---	---	0.722	0.3585	
Other	166558	502	0.30%	0.997	0.9532	
White	771164	2331	0.30%	1.000	---	
**Maternal Link**						
Yes	933959	3065	0.0033	0.949	0.949	
**Year**						
2010	214420	752	0.35%	1.070	0.1882	
2011	222591	751	0.34%	1.029	0.5737	
2012	229512	744	0.32%	0.989	0.8304	
2013	230730	733	0.32%	0.969	0.5462	
2014	233704	766	0.33%	1.000	---	
**Hispanic**						
Yes	701146	2082	0.30%	0.767	<.0001	
**Gestation age**						0.872
24-25 weeks	2103	553	20.82%	363.552	<.0001	
26-27 weeks	3085	257	7.69%	115.175	<.0001	
28-29 weeks	4282	183	4.10%	59.086	<.0001	
30-31 weeks	7160	164	2.24%	31.667	<.0001	
32-33 weeks	14868	207	1.37%	19.249	<.0001	
34-35 weeks	40567	280	0.69%	9.543	<.0001	
36-37 weeks	162043	397	0.24%	3.387	<.0001	
38-39 weeks	648416	469	0.07%	1.000	---	
40-41 weeks	241392	186	0.08%	1.065	0.4656	
42-43 weeks	5382	---	---	2.312	0.0128	
<24 weeks	676	1026	60.28%	---	<.0001	
>42 weeks	635	---	---	6.532	0.0012	
Unknown	348	12	3.33%	47.674	<.0001	

© 2024 Goodman DC et al. *JAMA Network Open.*

**Variable**	27 day death		Risk	Odds ratio	P-value	C statistic
**Brith Weight**	no	yes				0.875
1000-1099g	1332	72	5.13%	71.203	<.0001	
1100-1199g	1683	73	4.16%	57.135	<.0001	
1200-1299g	1522	60	3.79%	51.928	<.0001	
1300-1399g	2026	66	3.15%	42.911	<.0001	
1400-1499g	1975	64	3.14%	42.685	<.0001	
1500-1599g	2871	63	2.15%	28.905	<.0001	
1600-1699g	2646	54	2.00%	26.883	<.0001	
1700-1799g	3822	66	1.70%	22.747	<.0001	
1800-1899g	4889	79	1.59%	21.285	<.0001	
1900-1999g	4943	68	1.36%	18.121	<.0001	
2000-2099g	7707	68	0.87%	11.622	<.0001	
2100-2199g	7852	72	0.91%	12.079	<.0001	
2200-2299g	12818	93	0.72%	9.557	<.0001	
2300-2399g	14015	57	0.41%	5.357	<.0001	
2400-2499g	22214	83	0.37%	4.922	<.0001	
2500-2599g	24144	60	0.25%	3.273	<.0001	
2600-2699g	38904	72	0.18%	2.438	<.0001	
2700-2799g	42831	70	0.16%	2.153	<.0001	
2800-2899g	66418	74	0.11%	1.468	0.0171	
2900-2999g	66606	68	0.10%	1.345	0.0718	
3000-3099g	93642	78	0.08%	1.097	0.5588	
3100-3199g	83998	48	0.06%	0.753	0.1190	
3200-3299g	106697	81	0.08%	1.000	---	
3300-3399g	86437	49	0.06%	0.747	0.1067	
3400-3499g	97206	61	0.06%	0.827	0.2615	
3500-3599g	68673	39	0.06%	0.748	0.1366	
3600-3699g	70550	39	0.06%	0.728	0.1037	
3700-3799g	55352	26	0.05%	0.619	0.0332	
3800-3899g	36076	25	0.07%	0.913	0.6903	
3900-3999g	31590	14	0.04%	0.584	0.0630	

© 2024 Goodman DC et al. *JAMA Network Open.*

**Variable**	27 day death		Risk	Odds ratio	P-value	
**Brith Weight**	no	yes				
4000-4099g	18664	---	---	0.565	0.1231	
4100-4199g	16007	---	---	0.329	0.0301	
4200-4299g	9168	---	---	0.718	0.4730	
4300-4399g	7349	---	---	1.075	0.8636	
4400-4499g	4060	---	---	0.649	0.5458	
4500-4599g	3266	---	---	1.210	0.7459	
4600-4699g	1688	---	---	2.341	0.1483	
4700-4799g	1344	---	---	0.980	0.9841	
4800-4899g	753	---	---	3.499	0.0806	
4900-4999g	564	---	---	4.671	0.0316	
5000-5099g	310	0	0.00%	<0.001	0.9383	
5100-5199g	259	---	---	20.344	<.0001	
5200-5299g	108	---	---	12.197	0.0133	
5300-5399g	116	---	---	11.356	0.0162	
5400-5499g	67	---	---	19.660	0.0033	
5500-5599g	53	---	---	24.854	0.0016	
5600-5699g	37	0	0.00%	<0.001	0.9787	
5700-5799g	25	0	0.00%	<0.001	0.9825	
5800-5899g	13	0	0.00%	<0.001	0.9874	
400-499g	211	545	72.09%	---	<.0001	
500-599g	656	508	43.64%	---	<.0001	
600-699g	908	323	26.24%	468.580	<.0001	
700-799g	1223	226	15.60%	243.416	<.0001	
800-899g	1159	138	10.64%	156.842	<.0001	
900-999g	1507	120	7.38%	104.890	<.0001	
Unknown	---	0	---	<0.001	0.9939	

© 2024 Goodman DC et al. *JAMA Network Open.*

Variable	27 day death		Risk	Odds ratio	P-value	C statistic
**Education**	no	yes				0.506
8 years or fewer	91259	310	0.34%	0.960	0.5117	
College	343841	970	0.28%	0.797	<.0001	
Completed highschool	400015	1416	0.35%	1.000	---	
Some highschool	294987	1005	0.34%	0.962	0.3542	
Unknown	855	45	5.00%	14.868	<.0001	
**Multiple Birth**						0.548
Yes	29264	460	1.55%	5.270	<.0001	
**Smoking**						
Yes	95557	359	0.37%	1.149	0.0128	
**Maternal Link**						
Yes	933959	3065	0.33%	0.949	0.2204	
**Alcohol Use**						0.5
Yes	671	---	---	0.454	0.4302	
No	933289	3064	0.33%	1.000	---	
Unknown	196997	681	0.34%	1.053	0.2239	
**Diabetes**						
Yes	142130	487	0.34%	1.048	0.3400	
No	806141	2635	0.33%	1.000	---	
Unknown	182686	624	0.34%	1.045	0.3238	
**RHiso**						0.501
Yes	-9999	---	---	76.790	<.0001	
No	933928	3057	0.33%	1.000	---	
Unknown	196993	680	0.34%	1.055	0.2109	
**Breech**						0.615
Yes	83580	1136	1.34%	5.561	<.0001	
No	857048	2095	0.24%	1.000	---	
Unknown	190329	515	0.27%	1.107	0.0391	
**Cervix**						0.539
Yes	9976	326	3.16%	11.033	<.0001	
No	923997	2739	0.30%	1.000	---	
Unknown	196984	681	0.34%	1.166	0.0003	
**Chorioamnionitis**						0.542
Yes	29878	413	1.36%	4.717	<.0001	
No	905824	2655	0.29%	1.000	---	
Unknown	195255	678	0.35%	1.185	<.0001	
**Congenital**						0.648
Yes	53216	1283	2.35%	10.550	<.0001	

© 2024 Goodman DC et al. *JAMA Network Open.*

Variable	27 day death		Risk	Odds ratio	P-value	C statistic
**Cord Prolapse**	no	yes				0.527
Yes	139480	259	0.19%	0.526	<.0001	
No	794580	2806	0.35%	1.000	---	
Unknown	196897	681	0.34%	0.979	0.6266	
**Drug Use**						0.506
Yes	31846	147	0.46%	1.427	<.0001	
No	902281	2918	0.32%	1.000	---	
Unknown	196830	681	0.34%	1.070	0.1134	
**Fetal Distress**						0.517
Yes	57001	317	0.55%	1.765	<.0001	
No	879891	2772	0.31%	1.000	---	
Unknown	194065	657	0.34%	1.075	0.0978	
**Growth Restriction**						0.514
Yes	132531	547	0.41%	1.305	<.0001	
No	803690	2542	0.32%	1.000	---	
Unknown	194736	669	0.34%	1.067	0.1409	
**Hematologic**						0.511
Yes	10707	118	1.09%	3.443	<.0001	
No	925032	2961	0.32%	1.000	---	
Unknown	195218	667	0.34%	1.067	0.1287	
**Hypertension**						0.518
Yes	138352	592	0.43%	1.373	<.0001	
No	798430	2490	0.31%	1.000	---	
Unknown	194175	664	0.34%	1.097	0.0352	
**Infection**						0.522
Yes	228223	921	0.40%	1.329	<.0001	
No	705816	2144	0.30%	1.000	---	
Unknown	196918	681	0.34%	1.138	0.0032	
**Large for Age**						0.547
Yes	200041	310	0.15%	0.424	<.0001	
No	757649	2769	0.36%	1.000	---	
Unknown	200041	310	0.15%	1.053	0.2294	
**Oligohydramnios**						0.506
Yes	2923	57	1.91%	6.026	<.0001	
No	931464	3017	0.32%	1.000	---	
Unknown	196570	672	0.34%	1.055	0.2062	
**Placenta Abruption**						0.553
Yes	44629	547	1.21%	4.310	<.0001	
No	889707	2530	0.28%	1.000	---	
Unknown	196621	669	0.34%	1.197	<.0001	
**Placenta Previa**						0.505
Yes	27429	126	0.46%	1.417	<.0001	
No	906532	2939	0.32%	1.000	---	
Unknown	196996	681	0.34%	1.066	0.1319	
**Polyhydraminos**						0.507
Yes	934	56	5.66%	18.542	<.0001	
No	933138	3020	0.32%	1.000	---	
Unknown	196885	670	0.34%	1.051	0.2406	

© 2024 Goodman DC et al. *JAMA Network Open.*

**Variable**	27 day death		Risk	Odds ratio	P-value	C statistic
**Preeclampsia**	no	yes				
Yes	135581	529	0.39%	1.225	<.0001	
No	807918	2574	0.32%	1.000	---	
Unknown	187458	643	0.34%	1.077	0.0945	
**Preterm Labor**						0.649
Yes	215758	1648	0.76%	3.869	<.0001	
No	718284	1418	0.20%	1.000	---	
Unknown	196915	680	0.34%	1.749	<.0001	
**Prior Preterm**						0.541
Yes	45242	456	1.00%	3.394	<.0001	
No	892477	2651	0.30%	1.000	---	
Unknown	193238	639	0.33%	1.113	0.0151	
**Renal**						0.502
Yes	12050	53	0.44%	1.348	0.0317	
No	921911	3012	0.33%	1.000	---	
Unknown	196996	681	0.34%	1.058	0.184	
**Steroid**						0.528
Yes	27190	300	1.09%	3.575	<.0001	
No	911144	2812	0.31%	1.000	---	
Unknown	192623	634	0.33%	1.066	0.1438	
**Prenatal Care**						0.56
Yes	1071837	3097	0.29%	0.263	<.0001	
**Outborn**						0.549
Yes	13675	410	2.91%	10.043	<.0001	

© 2024 Goodman DC et al. *JAMA Network Open.*

From these analyses, it was decided to:

1. Combine Asian, Native American and Other race with White (p>0.1).
2. Drop Year as a variable (p>0.1 for 2010 and >0.5 for 2011-13.)
3. Combine Unknown with No for the following variables: Drug Use (p>0.10), Fetal Distress (p> 0.09), Growth Restriction (p>0.10), Hematologic (p>0.10), Oligohydramnios (p>0.20), Placenta Previa (p>0.10), Polyhydramnios (p>0.20), Preeclampsia (p> 0.09), Renal (p>0.10), Maternal Steroids (p>0.10).
4. Drop the following variables: Diabetes (all indicator variables p>0.30), Alcohol (all indicator variables p>0.20).

Two birth weight parameter sets were examined – 1) specified as 100 gram interval indicator variables (n=56), and 2) specified as a quartic function. (Goodman, et al. N Engl J Med 2002; Kiely J, et al. Statistics in Medicine. 1993). The quartic function was used as the model fit was slightly better and number of parameters was much smaller. (Appendix Table 7)

Appendix Table 7. Comparison of modelling birth weight as 100 gram indicator variables or as quartic function.

	BW as 100 gram increment indicator variables	BW as quartic function
Percent concordant	78.1	79.2
Percent discordant	3.1	3.6
Percent Tied	18.8	17.2
Somer's D	0.75	0.756
c	0.875	0.878
Number of parameters	56	4

<u>Multi-variable models</u>

The next variables that were tested were gestational age, growth restriction, large for gestational age, and preterm labor. In a model including birth weight, the added information provided by these variables would be expected to indicate the discordance of gestational age and birth weight in some newborns.

As seen in Appendix Table 8, gestational age as a 12-level indicator variable resulted in better mortality prediction than the other three variables.

Appendix Table 8. Birth weight and additional variables representing gestational age information.

	BW as quartic function	BW + GA is 12 indicator variables	BW + Growth Restriction	BW + Growth Restriction+Large for Gestational Age	BW + Preterm labor
Percent concordant	79.2	79.5	79.1	79.1	79.1
Percent discordant	3.6	3.5	3.6	3.6	3.5
Percent Tied	17.2	17	17.3	17.4	17.4
Somer's D	0.756	0.76	0.755	0.755	0.755
c	0.878	0.88	0.878	0.877	0.878
Number of parameters	4	17	6	8	7
P-value of Log Likelihood Ratio compare dto BW alone		<0.001	<0.001	<0.001	<0.001

With birth weight and gestational age included, each of the additional variables was added to derive a set of three variable models (i.e. birth weight terms, gestational age terms, and the additional variable under consideration).

After discussion with the study team clinicians, the following variables were included in all cohort models given the evidence of their association with neonatal mortality from previous literature- birth weight, gestational age, congenital anomaly, sex, education, race, and ethnicity.

The remaining variables were introduced simultaneously introduced and then variables were stepwise deleted to see effects on model likelihood ratio and C-statistic with the following results:

Four variables (indicated in red) appeared not to add additional information to the model and were deleted from further models – Drug Use, Hematologic, Placenta Previa, and Renal. The remaining variables were then simultaneously specified with the following results (Appendix Table 9):

© 2024 Goodman DC et al. *JAMA Network Open.*

Appendix Table 9. 27-day mortality multivariable final models for Cohort 1.

27-day Mortality Multivariable Final Model (Cohort 1)					
**Variable**	**Final Model**				
	Odds Ratio	95% Wald Confidence Interval		Wald Chi-Square	P-value
**Birth Weight**					
Birth weight	1.000	1.000	1.000	56.741	<.0001
Birth weight2	1.000	1.000	1.000	3.616	0.057
Birth weight3	1.000	1.000	1.000	0.937	0.333
Birth weight4	1.000	1.000	1.000	2.201	0.138
**Gestation age**					
24-25 weeks	2.423	1.808	3.247	35.115	<.0001
26-27 weeks	1.302	0.977	1.737	3.233	0.072
28-29 weeks	1.267	0.955	1.680	2.693	0.101
30-31 weeks	1.595	1.225	2.078	11.990	0.001
32-33 weeks	2.187	1.738	2.752	44.608	<.0001
34-35 weeks	2.530	2.102	3.046	96.264	<.0001
36-37 weeks	2.022	1.750	2.337	91.038	<.0001
38-39 weeks	1.000	1.000	1.000	1.000	1.000
40-41 weeks	1.263	1.062	1.502	6.989	0.008
42-43 weeks	2.630	1.355	5.107	8.162	0.004
<24 weeks	13.578	9.908	18.608	263.186	<.0001
>42 weeks	5.838	1.769	19.268	8.389	0.004
**Sex**					
Female	0.815	0.755	0.879	28.114	<.0001
**Race**					
Black	0.900	0.804	1.007	3.376	0.066
**Maternal Link**					
Yes	1.694	1.528	1.879	100.057	<.0001
**Hispanic**					
Yes	0.937	0.854	1.029	1.854	0.173
**Education**					
8 years or fewer	1.270	1.106	1.460	11.403	0.001
College	0.708	0.649	0.774	59.064	<.0001
Completed highschool	1.000	1.000	1.000	1.000	1.000
**Multiple Birth**					
Yes	0.707	0.627	0.798	31.391	<.0001
**Breech**					
Yes	1.442	1.317	1.580	62.107	<.0001
**Congenital**					
Yes	3.189	2.915	3.489	638.198	<.0001
**Cord Prolapse**					
Yes	0.717	0.621	0.829	20.332	<.0001
**Fetal Distress**					
Yes	1.328	1.157	1.524	16.303	<.0001
**Hypertension**					
Yes	0.561	0.505	0.623	116.874	<.0001
**Oligohydramnios**					
Yes	2.937	2.159	3.995	47.059	<.0001
**Polyhydraminos**					
Yes	14.918	10.813	20.580	270.927	<.0001
**Steroid**					
Yes	0.599	0.519	0.691	49.335	<.0001
**Outborn**					
Yes	0.761	0.666	0.870	16.074	<.0001
**Placenta Abruption**					
Yes	1.150	1.022	1.295	5.390	0.020
**RH Isoimmunization**					
Yes	17.238	7.020	42.324	38.591	<.0001

© 2024 Goodman DC et al. *JAMA Network Open.*

Overall the model statistics were favorable with a c of 0.912 and a Somers' D of 0.824.

<u>Sensitivity testing</u>

Deletion of variables in varying order did not alter the final model specification.

The final model parameters were then used to predict 60 and 90-day mortality. As expected, for later periods of mortality, the model fit decreased (c statistic 0.903 for 60 day mortality and 0.886 for 90 day mortality) reflecting the non-perinatal causes of death, including the beneficial effects of medical care, that are reflected in these time periods.

Remaining cohorts

The models for the remaining cohorts were developed using the same procedures for Cohort 1. Final model specifications are seen in Appendix Tables 10.

Appendix Table 10. Final model specification by Cohorts. Note that this study includes only Cohorts, 3 (very low birth weight singleton), 5 (late preterm singleton), and 6 (non-preterm singletons).

	Final Model Variables by Cohort					
	**Cohort 1**	**Cohort 2**	**Cohort 3**	**Cohort 4**	**Cohort 5**	**Cohort 6**
	Birth weight4	Birth weight4	Birth weight3	Birth weight3	Birth weight4	Birth weight4
	Congenital	Congenital	Congenital	Congenital	Congenital	Congenital
	Gestational Age	Gestational Age	Gestational Age	Gestational Age	Gestational Age	Gestational Age
	Sex	Sex	Sex	Sex	Sex	Sex
	Race	Race	Race	Race	Race	Race
	Hispanic	Hispanic	Hispanic	Hispanic	Hispanic	Hispanic
	Polyhydramnios	Polyhydramnios	Polyhydramnios		Polyhydramnios	Polyhydramnios
	Hypertension	Hypertension	Hypertension	Hypertension	Hypertension	
	Breech	Breech	Breech		Breech	Breech
	Maternal Link	Maternal Link	Maternal Link	Maternal Link	Maternal Link	Maternal Link
	Outborn	Outborn	Outborn	Outborn	Outborn	Outborn
	Education	Education	Education	Education	Education	Education
	Fetal Distress	Fetal Distress				Fetal Distress
	Oligohydramnios	Oligohydramnios	Oligohydramnios		Oligohydramnios	
	Steroid	Steroid	Steroid	Steroid		
	Cord Prolapse	Cord Prolapse	Cord Prolapse	Cord Prolapse		
			Hematologic	Hematologic		
	Placenta Abruption					
	RH Isoimmunization	RH Isoimmunization			RH Isoimmunization	
	Multiple Birth					
				Prior Preterm		
			Preeclampsia			
Total Variables	19	17	17	14	14	12
DF	33	31	24	21	19	19
Somers' D	0.824	0.82	0.713	0.726	0.737	0.565
C-statistic	0.912	0.91	0.856	0.863	0.868	0.782

Note: Hypertension refers to maternal hypertension during pregnancy.

© 2024 Goodman DC et al. *JAMA Network Open.*

Step 2. Method used to calculate adjusted risk ratios and rates of newborn care events across hospitals:

To account for illness associated with a low probability of mortality, but still generally requiring medical care, we tabulated diagnoses and major procedures across all cohorts and across varying lengths of stay. From this, study neonatologists and pediatricians identified single lists of 1) diagnoses that would require NICU care in some infants, 2) congenital anomalies requiring NICU care in some infants (note that this is different than the list used to predict mortality) and 3) major procedures. (Appendix Tables 11, 12, 13). Each newborn was then coded with a flag indicating the 1) presence of any listed diagnosis, 2) the presence of any congenital anomaly and 2) presence of any listed major procedure.

© 2024 Goodman DC et al. *JAMA Network Open.*

Appendix Table 11. Diagnoses used in risk adjustment Model 2.

Diagnosis Code	Diagnosis Description	Category
77081	PRIMARY APNEA OF NEWBORN	
77082	OTHER APNEA OF NEWBORN	Apnea
7454	VENTRICULAR SEPTAL DEFECT	
7455	OSTIUM SECUNDUM AT/SEPTAL DEFECT	
7470	PATENT DUCTUS ARTERIOSUS	
7473	ANOMALIES PULMONARY ARTERY	
7852	UNDIAGNOSED CARDIAC MURMURS	
74602	STENOSIS PULMONARY VALVE CONGENITAL	
74689	OTHER ANOMALIES HEART	
74710	COARCTATION OF AORTA	
74731	PULM ARTERY COARCTATION AND ATRESIA	
74783	PERSISTENT FETAL CIRCULATION	
77981	NEONATAL BRADYCARDIA	
77982	NEONATAL TACHYCARDIA	Cardiac
77083	CYANOTIC ATTACKS OF NEWBORN	Cyanosis
77931	NEWBORN FEEDING PROBLEMS	Feeding
7756	NEONATAL HYPOGLYCEMIA	Hypoglycemia
7783	OTH HYPOTHERMIA NEWBORN	
7784	OTH TEMPERATURE REGULATION DISTURB	Hypothermia
7750	SYNDROME INFANT A DIABETIC MOTHER	IDM
4104	STREPTOCOCCUS GROUP D INFECTION	
4185	OTH GRAM NEGATIVE ORG INFECTION	
77181	SEPTICEMIA OF NEWBORN	
99592	SEVERE SEPSIS	Infection
77210	INTRAVENT HEMORRAGE UNSP GRADE	
77211	INTRAVENT HEMORRHAGE GRADE I	
77212	INTRAVENT HEMORRAGE GRADE II	
77213	INTRAVENT HEMORRAGE GRADE III	
77214	INTRAVENT HEMORRAGE GRADE IV	IVH
7731	NEWBORN HEMOLYT DIS ABO ISOIM	
7732	NEWBORN HEMOLYT DIS ISOIM OT	
7742	NEONATAL JAUNDICE PRETERM DEL	
7746	FETAL/NEONATAL JAUNDICE UNSPEC	Jaundice
76384	MECONIUM PASSAGE DUR DELIVER	
77984	MECONIUM STAINING	Meconium
2760	HYPEROSMOLALITY/OR HYPERNATREMIA	
2761	HYPOSMOLALITY/OR HYPONATREMIA	
2762	ACIDOSIS	
7754	HYPOCALCEMIA HYPOMAGNESEMIA NEWBORN	
7755	OTH NEONATAL ELECTROLYTE DISTURBANC	
7757	LATE METABOLIC ACIDOSIS NEWBORN	
77581	OTHER ACIDOSIS OF NEWBORN	Metabolic
7795	NEWBORN DRUG WITHDRAWAL SYNDR	NAS
7424	OTHER ANOMALIES BRAIN	
7790	CONVULSIONS IN NEWBORN	Neurologic
769	RESPIRATORY DISTRESS SYNDROME NB	
7485	AGENESIS HYPOPLASIA/DYSPLASIA LUNG	
7700	CONGENITAL PNEUMONIA	
7702	INTERSTITIAL EMPHYSEMA PERINATAL	
7704	PRIMARY ATELECTASIS PERINATAL	
7705	OTH UNS ATELECTASIS PERINATAL	
7706	TRANSITORY TACHYPNEA NEWBORN	
77084	RESPIRATORY FAILURE OF NEWBORN	
77089	OT RESPIRATORY PROBLEM AFTER BIRTH	Respiratory

© 2024 Goodman DC et al. *JAMA Network Open.*

Appendix Table 12. Congenital anomalies associated with increased likelihood of NICU care used in risk adjustment model 2.

**icd9**	**Label**
65500	Anencephaly
7419	Spina bifida without mention hydrocephalus, unspecified region
7423	Congenital hydrocephalus
7423	Congenital hydrocephalus
7422	Reduction deformaties of brain
7420	Encephalocele
7450	Common truncus
74510	Complete transposition of great vessels
7452	Tetrology of Fallot
7453	Common ventricle
74511	Double outlet right ventricle (DORV)
74569	Other
74601	Atresia, congenital
7461	Tricuspid atresia and stenosis, congenital
7467	Hypoplastic left heart syndrome
74711	Interruption of aortic arch
74741	Total anomalous pulmonary venous
75989	Pentalogy of Cantrell (Thoraco-Abdominal Ectopia Cordis)
74710	Coarctation of aorta (preductal) (postductal)
74710	Arrythmia requiring surgical or medical intervention
7462	Ebstein's anomaly
74602	Pulmonary Valvular Stenosis requiring surgical or medical intervention
7503	Tracheoesophageal fistula, esophageal atresia and stenosis
7503	Tracheoesophageal fistula, esophageal atresia and stenosis
7511	Atresia and stenosis of small intestine
7511	Atresia and stenosis of small intestine
7511	Atresia and stenosis of small intestine
7512	Atresia and stenosis of large intestine, rectum, and anal canal
7515	Other anomalies of intestine
75672	Omphalocele
75672	Gastroschisis
75673	Gastroschisis
75161	Biliary atresia
7513	Hirschsprung's disease and other congenital functional disorders of colon
2380	Sacrococcygeal teratoma requiring surgical or medical intervention
7530	Renal agenesis and dysgenesis

© 2024 Goodman DC et al. *JAMA Network Open.*

75310	Cystic kidney disease, unspecified
75320	Unspecified obstructive defect of renal pelvis and ureter
7535	Exstrophy, bladder
7581	Trisomy 13
7582	Trisomy 18
7566	Anomalies of diaphragm
7780	Hydrops Fetalis with anasarca and one or more of the following: ascites, pleural effusion, pericardial effusion
7612	Oligohydramnios sequence
35922	Myotonic Dystrophy requiring endotracheal intubation and assisted ventilation
7594	Conjoined twins
7483	Other anomalies of larynx, trachea, and bronchus
74259	Thanatophoric Dysplasia Types 1 and 2
28243	Hemoglobin Barts
7623	Twin - twin transfusion syndrome
7484	Congenital cystic lung
4251	Hypertrophic obstructive cardiomyopathy
5533	Diaphragmatic hernia
5602	Volvulus
7400	Anencephalus
7401	Anencephalus
7410	Spina bifida without anencephalus
74512	Corrected transposition of great vessels
74519	Other
74560	Endocardial cushion defect, unspecified type
74602	Pulmonary valve atresia and stenosis
7463	Congenital stenosis of aoirtic valve
7465	Congenital mitral stenosis
7471	Coarctation of aorta
74722	Atresia and stenosis of aorta
74742	Partial anomalous pulmonary venous connection
75314	Polycystic kidney, autosomal recessive
75321	Congenital obstruction of ureteropelvic junction
75322	Congenital obstruction of ureterovesical junction
7536	Atresia and stenosis of urethra and bladder neck
75651	Osteogenesis imperfecta
75671	Prune Belly Syndrome
7597	Multiple congenital anomalies
7780	Hydrops getalis not due to isoimmunization

© 2024 Goodman DC et al. *JAMA Network Open.*

Appendix Table 13. Major procedures used in risk adjustment Model 2.

Procedure Code	Procedure Description
64	COMPLETE THYROIDECTOMY
212	OTHER REPAIR OF CEREBRAL MENINGES
222	INTRACRANIAL VENTRICULAR SHUNT OR ANASTOMOSIS
234	VENTRICULAR SHUNT TO ABDOMINAL CAVITY AND ORGANS
341	INCISION OF MEDIASTINUM
351	REPAIR OF SPINAL MENINGOCELE
352	REPAIR OF SPINAL MYELOMENINGOCELE
390	SYSTEMIC TO PULMONARY ARTERY SHUNT
461	COLOSTOMY
462	ILEOSTOMY
2189	OTHER REPAIR AND PLASTIC OPERATIONS ON NOSE
2559	OTHER REPAIR AND PLASTIC OPERATIONS ON TONGUE
3173	CLOSURE OF OTHER FISTULA OF TRACHEA
3201	ENDOSCOPIC EXCISION OR DESTRUCTION OF LESION OR TISSUE OF BRONCHUS
3479	OTHER REPAIR OF CHEST WALL
3514	OPEN HEART VALVULOPLASTY OF TRICUSPID VALVE WITHOUT REPLACEMENT
3541	ENLARGEMENT OF EXISTING ATRIAL SEPTAL DEFECT
3542	CREATION OF SEPTAL DEFECT IN HEART
3552	REPAIR OF ATRIAL SEPTAL DEFECT WITH PROSTHESIS, CLOSED TECHNIQUE
3562	REPAIR OF VENTRICULAR SEPTAL DEFECT WITH TISSUE GRAFT
3571	OTHER AND UNSPECIFIED REPAIR OF ATRIAL SEPTAL DEFECT
3582	TOTAL REPAIR OF TOTAL ANOMALOUS PULMONARY VENOUS CONNECTION
3584	TOTAL CORRECTION OF TRANSPOSITION OF GREAT VESSELS, NOT ELSEWHERE CLASSIFIED
3592	CREATION OF CONDUIT BETWEEN RIGHT VENTRICLE AND PULMONARY ARTERY
3723	COMBINED RIGHT AND LEFT HEART CARDIAC CATHETERIZATION
3834	RESECTION OF VESSEL WITH ANASTOMOSIS, AORTA
3845	RESECTION OF VESSEL WITH REPLACEMENT, THORACIC VESSELS
3885	OTHER SURGICAL OCCLUSION OF VESSELS, THORACIC VESSELS
3961	EXTRACORPOREAL CIRCULATION AUXILIARY TO OPEN HEART SURGERY
3965	EXTRACORPOREAL MEMBRANE OXYGENATION [ECMO]
4285	REPAIR OF ESOPHAGEAL STRICTURE
4466	OTHER PROCEDURES FOR CREATION OF ESOPHAGOGASTRIC SPHINCTERIC COMPETENCE
4526	OPEN BIOPSY OF LARGE INTESTINE
4533	LOCAL EXCISION OF LESION OR TISSUE OF SMALL INTESTINE, EXCEPT DUODENUM
4561	MULTIPLE SEGMENTAL RESECTION OF SMALL INTESTINE
4562	OTHER PARTIAL RESECTION OF SMALL INTESTINE
4579	OTHER AND UNSPECIFIED PARTIAL EXCISION OF LARGE INTESTINE
4591	SMALL-TO-SMALL INTESTINAL ANASTOMOSIS
4603	EXTERIORIZATION OF LARGE INTESTINE
4610	COLOSTOMY, NOT OTHERWISE SPECIFIED
4611	TEMPORARY COLOSTOMY
4620	ILEOSTOMY, NOT OTHERWISE SPECIFIED
4621	TEMPORARY ILEOSTOMY
4639	OTHER ENTEROSTOMY
4681	INTRA-ABDOMINAL MANIPULATION OF SMALL INTESTINE
4682	INTRA-ABDOMINAL MANIPULATION OF LARGE INTESTINE
4709	OTHER APPENDECTOMY
4719	OTHER INCIDENTAL APPENDECTOMY
4840	PULL-THROUGH RESECTION OF RECTUM, NOT OTHERWISE SPECIFIED
5372	OTHER AND OPEN REPAIR OF DIAPHRAGMATIC HERNIA, ABDOMINAL APPROACH
5383	LAPAROSCOPIC REPAIR OF DIAPHRAGMATIC HERNIA, WITH THORACIC APPROACH
5411	EXPLORATORY LAPAROTOMY
5419	OTHER LAPAROTOMY
5421	LAPAROSCOPY
5459	OTHER LYSIS OF PERITONEAL ADHESIONS
5471	REPAIR OF GASTROSCHISIS
5493	CREATION OF CUTANEOPERITONEAL FISTULA
5495	INCISION OF PERITONEUM
5721	VESICOSTOMY
5789	OTHER REPAIR OF BLADDER

© 2024 Goodman DC et al. *JAMA Network Open.*

Risk ratios for study measures were estimated using the following GEE Poisson models with clustering by hospital-year.

These models were specified similarly. The unit of analysis was the newborn. The dependent variables were the utilization or outcome measures. The independent variables were: 1) indicator variables for capacity-year with the lowest value category serving as the reference group 2) indicator variable for categories of newborn predicted 27 day mortality, 3) indicator variable for presence of a diagnosis 4) indicator variable for presence of a congenital anomaly 5) indicator variable for presence of a major procedure.

Capacity was specified for each year as a continuous and categorical variable in separate models, with similar findings. For reporting findings, we used capacity categories which are simpler to interpret. Categories of capacity, which are presented in paper Tables 3 and 4 were selected to represent reasonable levels of capacity, as per study investigators, and numbers of newborns and hospitals.

The distribution of predicted mortality was highly skewed and differed for each cohort. Since model fit was poor when predicted mortality was specified as a continuous variable, we explored several nonlinear expressions. Various categories were assessed for clinical reasonableness and statistical characteristics, such as sufficient newborns in each category and model fit and discrimination. Categorizing predicted risk by doubling of predicted mortality produced a measure with face validity to study neonatologists, provided sufficient numbers of newborns in each category, and had good model discrimination and goodness of fit. Examples of the categories are seen in Appendix Tables 14 – 16.

Appendix Table 14. Cohort 1 (live births ≥400 grams) predicted 27 day mortality distribution across categories used in Model 2.

Category	Live Births	Predicted Mortality	Mean Predicted Mortality	Std Dev	Minimum	Maximum
1	337429	**<0.0005**	0.0004	0.0001	0.0001	0.0005
2	455510	**0.0005 to <0.001**	0.0007	0.0001	0.0005	0.0010
3	186089	**0.001 to <0.002**	0.0014	0.0003	0.0010	0.0020
4	81407	**0.002 to <0.004**	0.0028	0.0006	0.0020	0.0040
5	35403	**0.004 to <0.008**	0.0055	0.0011	0.0040	0.0080
6	16969	**0.008 to <0.016**	0.0111	0.0022	0.0080	0.0160
7	8645	**0.016 to <0.032**	0.0222	0.0045	0.0160	0.0320
8	4728	**0.032 to <0.064**	0.0446	0.0090	0.0320	0.0640
9	2843	**0.064 to <0.128**	0.0899	0.0181	0.0640	0.1280
10	1861	**0.128 to <0.256**	0.1810	0.0366	0.1280	0.2558
11	1160	**0.256 to <0.512**	0.3634	0.0747	0.2561	0.5119
12	1397	**0.512 to <1.0**	0.6881	0.1010	0.5121	0.9487

Appendix Table 15 Cohort 3 (very low birth weight singleton) predicted 27 day mortality distribution across categories used in Model 2.

Category	Live Births	Predicted Mortality	Mean Predicted Mortality	Std Dev	Minimum	Maximum
1	626	**<0.01**	0.0071	0.0020	0.0009	0.0100
2	1580	**0.01 to <0.02**	0.0151	0.0029	0.0100	0.0200
3	3000	**0.02 to <0.04**	0.0295	0.0058	0.0200	0.0400
4	3051	**0.04 to <0.08**	0.0568	0.0112	0.0400	0.0800
5	1875	**0.08 to <0.16**	0.1122	0.0227	0.0801	0.1600
6	1085	**0.16 to <0.32**	0.2240	0.0451	0.1600	0.3196
7	808	**0.32 to <0.64**	0.4604	0.0931	0.3201	0.6380
8	801	**0.64 to <1.00**	0.8000	0.0866	0.6403	0.9566

Appendix Table 16. Cohort 5 (late preterm singleton) predicted 27 day mortality distribution across categories used in Model 2.

Category	Live Births	Predicted Mortality	Mean Predicted Mortality	Std Dev	Minimum	Maximum
1	13438	**<0.001**	0.0008	0.0001	0.0003	0.0010
2	38869	**0.001 to <0.002**	0.0014	0.0003	0.0010	0.0020
3	14860	**0.002 to <0.004**	0.0027	0.0005	0.0020	0.0040
4	3508	**0.004 to <0.008**	0.0053	0.0011	0.0040	0.0080
5	1608	**0.008 to <0.016**	0.0117	0.0023	0.0080	0.0160
6	3055	**0.016 to <0.032**	0.0233	0.0043	0.0160	0.0320
7	1730	**0.032 to <0.064**	0.0441	0.0087	0.0320	0.0640
8	634	**0.064 to <0.128**	0.0877	0.0182	0.0642	0.1276
9	311	**0.128 to <1.00**	0.2217	0.1157	0.1281	0.8141

Nevertheless, the model of inpatient mortality for non-preterm newborns would not converge with predicted mortality specified as categories, and so was specified as a continuous variable.

Finally, model coefficients (Poisson) were exponentiated to produce adjusted risk ratios.

© 2024 Goodman DC et al. *JAMA Network Open.*

References. Literature identified for risk adjustment models

1. Aliaga S, Boggess K, Ivester T, Price W. Influence of Neonatal Practice Variation on Outcomes of Late Preterm Birth. *Am J Perinatol*. 2013;31(08):659-666. doi:10.1055/s-0033-1356484.

2. Alleman BW, Bell EF, Li L, et al. Individual and center-level factors affecting mortality among extremely low birth weight infants. *Pediatrics*. 2013;132(1):e175-e184.

3. Attar MA, Lang SW, Gates MR, Iatrow AM, Bratton SL. Back transport of neonates: effect on hospital length of stay. *J Perinatol*. 2005;25(11):731-736.

4. Bender GJ, Koestler D, Ombao H, et al. Neonatal intensive care unit: predictive models for length of stay. *J Perinatol Off J Calif Perinat Assoc*. 2013;33(2):147-153. doi:10.1038/jp.2012.62.

5. Bronstein JM, Ounpraseuth S, Jonkman J, et al. Improving perinatal regionalization for preterm deliveries in a Medicaid covered population: initial impact of the Arkansas ANGELS intervention. *Health Serv Res*. 2011;46(4):1082-1103.

6. Centers for Disease Control and Prevention, Barfield, W.D. Neonatal intensive-care unit admission of infants with very low birth weight --- 19 States, 2006. *MMWR - Morb Mortal Wkly Rep*. 2010;59(44):1444-1447.

7. Chung JH, Phibbs CS, Boscardin WJ, Kominski GF, Ortega AN, Needleman J. The effect of neonatal intensive care level and hospital volume on mortality of very low birth weight infants. *Med Care*. 2010;48(7):635-644.

8. Chung JH, Phibbs CS, Boscardin WJ, et al. Examining the effect of hospital-level factors on mortality of very low birth weight infants using multilevel modeling. *J Perinat*. 2011;31(12):770-775.

9. Cooper WO, Hickson GB, Mitchel EF Jr, Ray WA. Comparison of perinatal outcomes among TennCare managed care organizations. *Pediatrics*. 1999;104(3 Pt 1):525-529.

10. Craighead DV, Elswick Jr. RK. The Influence of Early-Term Birth on NICU Admission, Length of Stay, and Breastfeeding Initiation and Duration. *J Obstet Gynecol Neonatal Nurs*. 2014;43(4):409-421. doi:10.1111/1552-6909.12472.

11. Freedman S. Capacity and Utilization in Health Care: The Effect of Empty Beds on Neonatal Intensive Care Admission. *J-Econ Policy*. 2016;8(2):154-185. doi:10.1257/pol.20120393.

12. Goodman DC, Fisher ES, Little GA, Stukel TA, Chang CH, Schoendorf KS. The relation between the availability of neonatal intensive care and neonatal mortality. *N Engl J Med*. 2002;346(20):1538-1544.

13. Gould JB, Qin C, Marks AR, Chavez G. Neonatal mortality in weekend vs weekday births. *JAMA*. 2003;289(22):2958-2962.

© 2024 Goodman DC et al. *JAMA Network Open.*

14. Haberland CA, Phibbs CS, Baker LC. Effect of opening midlevel neonatal intensive care units on the location of low birth weight births in California. *Pediatrics*. 2006;118(6):e1667-e1679. doi:10.1542/peds.2006-0612.

15. Hagen EW, Sadek-Badawi M, Albanese A, Palta M. A comparison of Wisconsin neonatal intensive care units with national data on outcomes and practices. *WMJ*. 2008;107(7):320-326.

16. Hintz SR, Bann CM, Ambalavanan N, et al. Predicting time to hospital discharge for extremely preterm infants. *Pediatrics*. 2010;125(1):e146-e154. doi:10.1542/peds.2009-0810.

17. Jensen EA, Lorch SA. Effects of a Birth Hospital's Neonatal Intensive Care Unit Level and Annual Volume of Very Low-Birth-Weight Infant Deliveries on Morbidity and Mortality. *JAMA Pediatr*. 2015;169(8):e151906. doi:10.1001/jamapediatrics.2015.1906.

18. Kastenberg ZJ, Lee HC, Profit J, Gould JB, Sylvester KG. Effect of Deregionalized Care on Mortality in Very Low-Birth-Weight Infants With Necrotizing Enterocolitis. *JAMA Pediatr*. November 2014. doi:10.1001/jamapediatrics.2014.2085.

19. Lapcharoensap W, Gage SC, Kan P, et al. Hospital Variation and Risk Factors for Bronchopulmonary Dysplasia in a Population-Based Cohort. *JAMA Pediatr*. 2015;169(2):e143676. doi:10.1001/jamapediatrics.2014.3676.

20. Lau C, Ambalavanan N, Chakraborty H, Wingate MS, Carlo WA. Extremely low birth weight and infant mortality rates in the United States. *Pediatrics*. 2013;131(5):855-860.

21. Lee HC, Bennett MV, Schulman J, Gould JB. Accounting for variation in length of NICU stay for extremely low birth weight infants. *J Perinatol*. 2013;33(11):872-876. doi:10.1038/jp.2013.92.

22. Lorch SA, Baiocchi M, Ahlberg CE, Small DS. The differential impact of delivery hospital on the outcomes of premature infants. *Pediatrics*. 2012;130(2):270-278.

23. Lorch SA, Passarella M, Zeigler A. Challenges to Measuring Variation in Readmission Rates of Neonatal Intensive Care Patients. *Acad Pediatr*. 2014;14(5, Supplement):S47-S53. doi:10.1016/j.acap.2014.06.010.

24. Ounpraseuth S, Gauss CH, Bronstein J, Lowery C, Nugent R, Hall R. Evaluating the effect of hospital and insurance type on the risk of 1-year mortality of very low birth weight infants: controlling for selection bias. *Med Care*. 2012;50(4):353-360.

25. Phibbs, C. S., L. C. Baker, A. B. Caughey, B. Danielsen, S. K. Schmitt, and R. H. Phibbs. "Level and Volume of Neonatal Intensive Care and Mortality in Very-Low-Birth-Weight Infants." *N Engl J Med* 356, no. 21 (2007): 2165–75.

© 2024 Goodman DC et al. *JAMA Network Open.*

eFigure 1. Texas Medicaid Newborn Study Cohort, 2010-2014

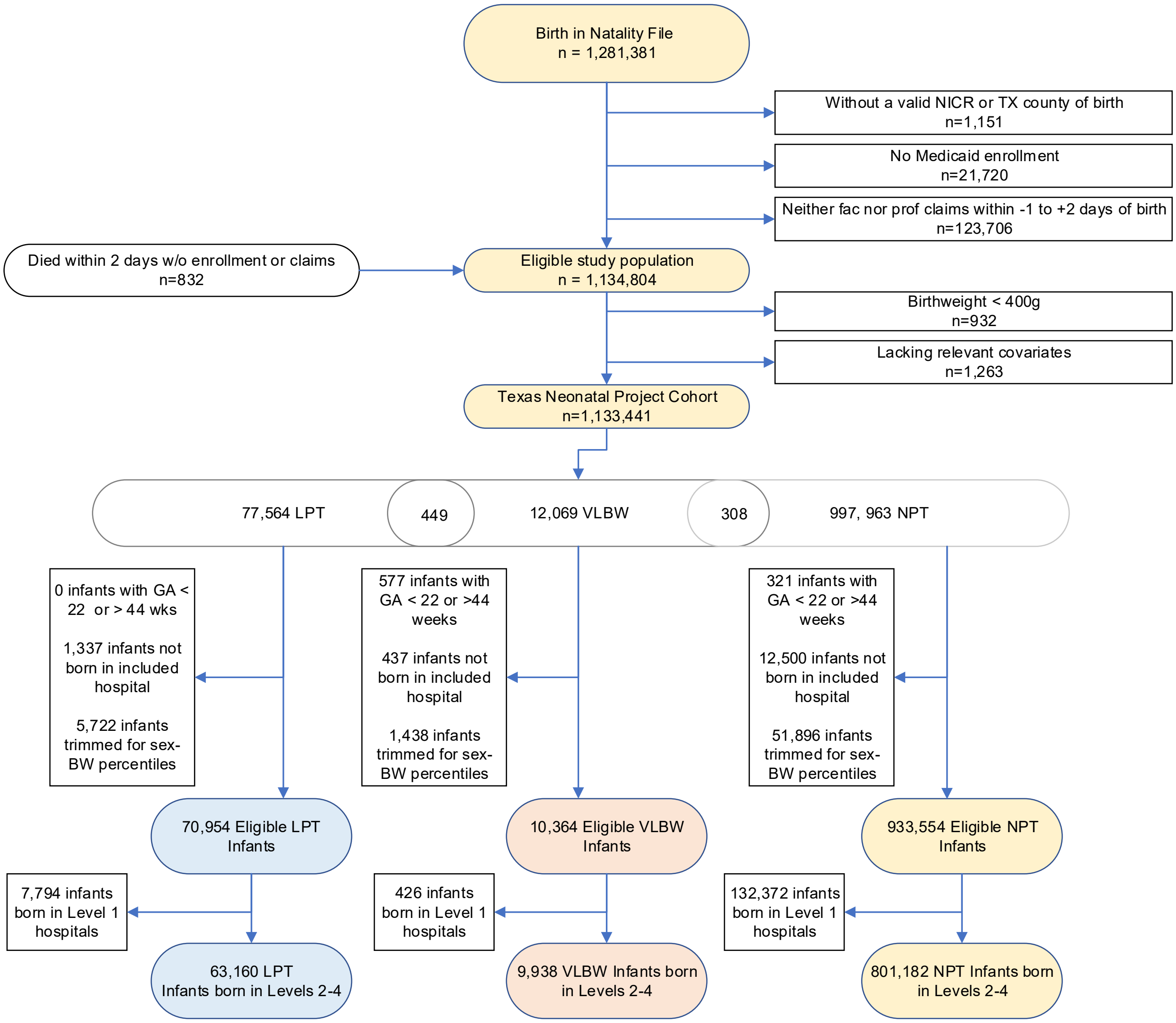

© 2024 Goodman DC et al. *JAMA Network Open.*

eFigure 2. NICU Bed Count Allocation Method

NICU bed exposure is intended to represent the beds available to neonates born in a hospital. For hospitals that transfer newborns in, the physical NICU beds occupied by these transferred-in newborns are “allocated” back to the birth hospital since the beds are available to their newborns. The receiving hospitals’ NICU beds are then reduced by the number of beds used by newborns transferred in. For hospitals that transfer newborns out, their “allocated” NICU bed count comprises their own NICU bed count plus the NICU beds used in other hospitals by their transferred newborns. This method “allocates” NICU beds to the hospital where babies are born by adjusting for beds used by newborns who are transferred in or transferred out.

The NICU beds that are “allocated” in or out of a birth hospital are measured by special care bed days for each transferred neonate by dividing the number of special care days by 365 days.

For each hospital:

Allocated NICU beds per live birth =

$$\frac{\text{reported beds – minus beds for newborns transferred in + beds for newborns transferred out}}{\text{Number of hospital births}}$$

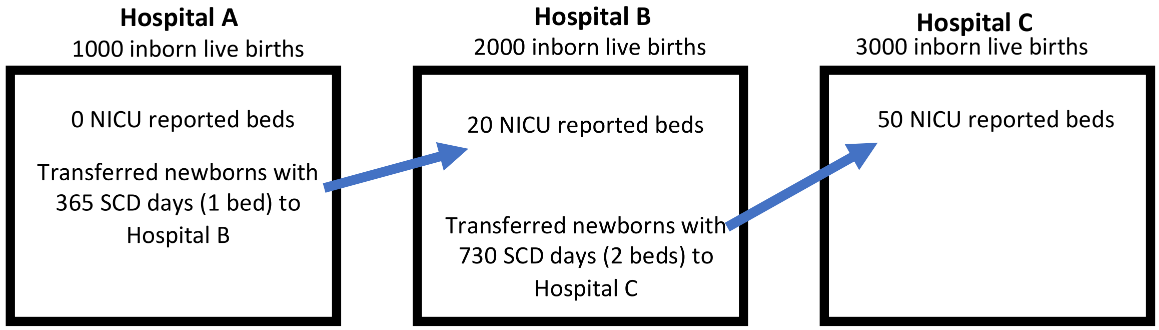

Allocated beds = 0 reported +1 for out transfers to hospital B

Allocated bed supply = 1 per 1000 LB

Allocated beds = 20 reported -1 for in transfers from hospital A + 2 for out transfers to hospital C

Allocated bed supply = 21 per 2000 LB = 10.5 per 1,000

Allocated beds = 50 reported -2 for in transfers from hospital B

Allocated bed supply = 48 per 3000 LB = 16 per 1,000

The total number of beds DOES NOT CHANGE. For each 365 days of SCD in newborns transferred out, the sending hospital’s NICU bed number is increased by 1 and the receiving hospital’s NICU bed number is decreased by 1.

© 2024 Goodman DC et al. *JAMA Network Open.*

eFigure 3. Associations of Hospital Allocated Neonatal Intensive Care Beds per Live Birth With Utilization, Stratified by Median Hospital Total Live Births, Texas Medicaid-Insured Newborns, 2010-2014

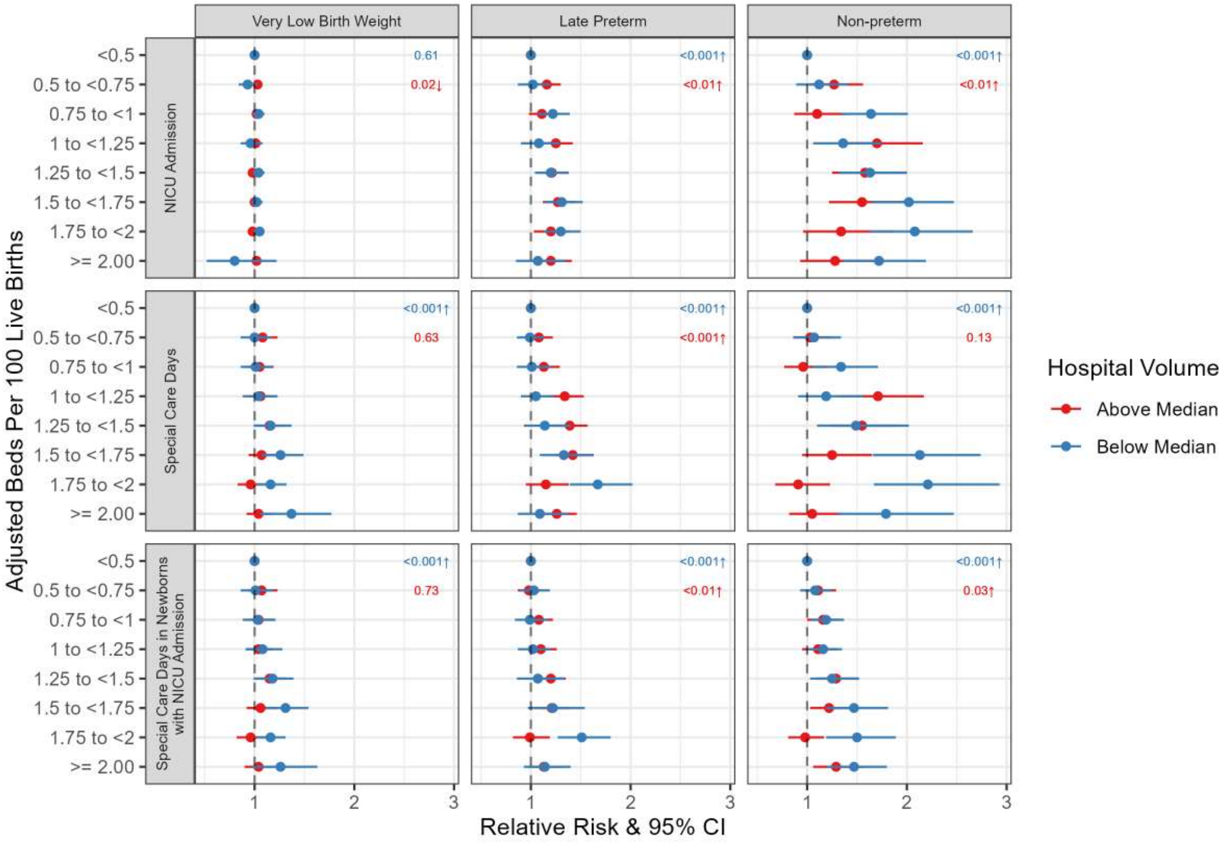

© 2024 Goodman DC et al. *JAMA Network Open.*

eFigure 4. Associations of Hospital Allocated Neonatal Intensive Care Beds per Live Birth With Utilization, Stratified by Hospital Profit Status, Texas Medicaid-Insured Newborns, 2010-2014

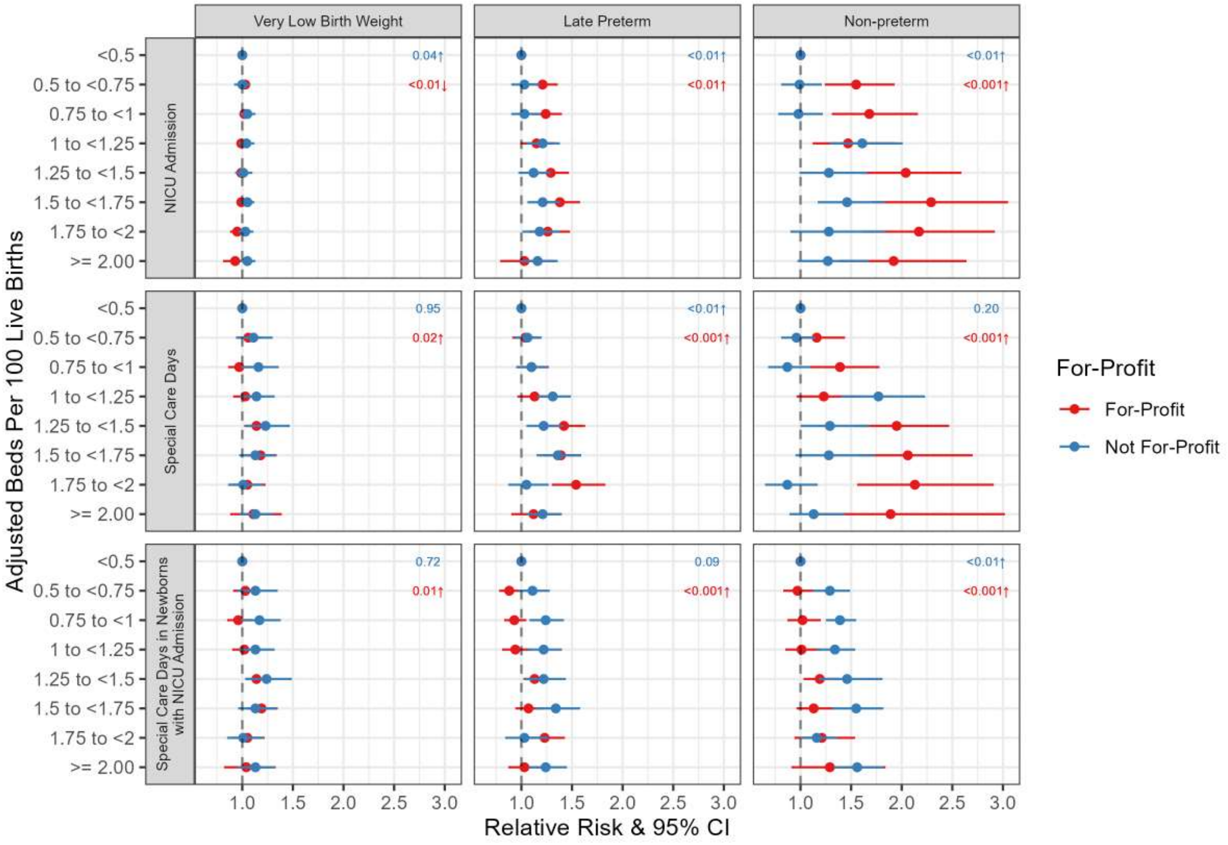

© 2024 Goodman DC et al. *JAMA Network Open.*

eFigure 5. Associations of Hospital Allocated Neonatal Intensive Care Beds per Live Birth With Utilization, Stratified by Presence of Hospital Neonatal Fellowship, Texas Medicaid-Insured Newborns, 2010-2014

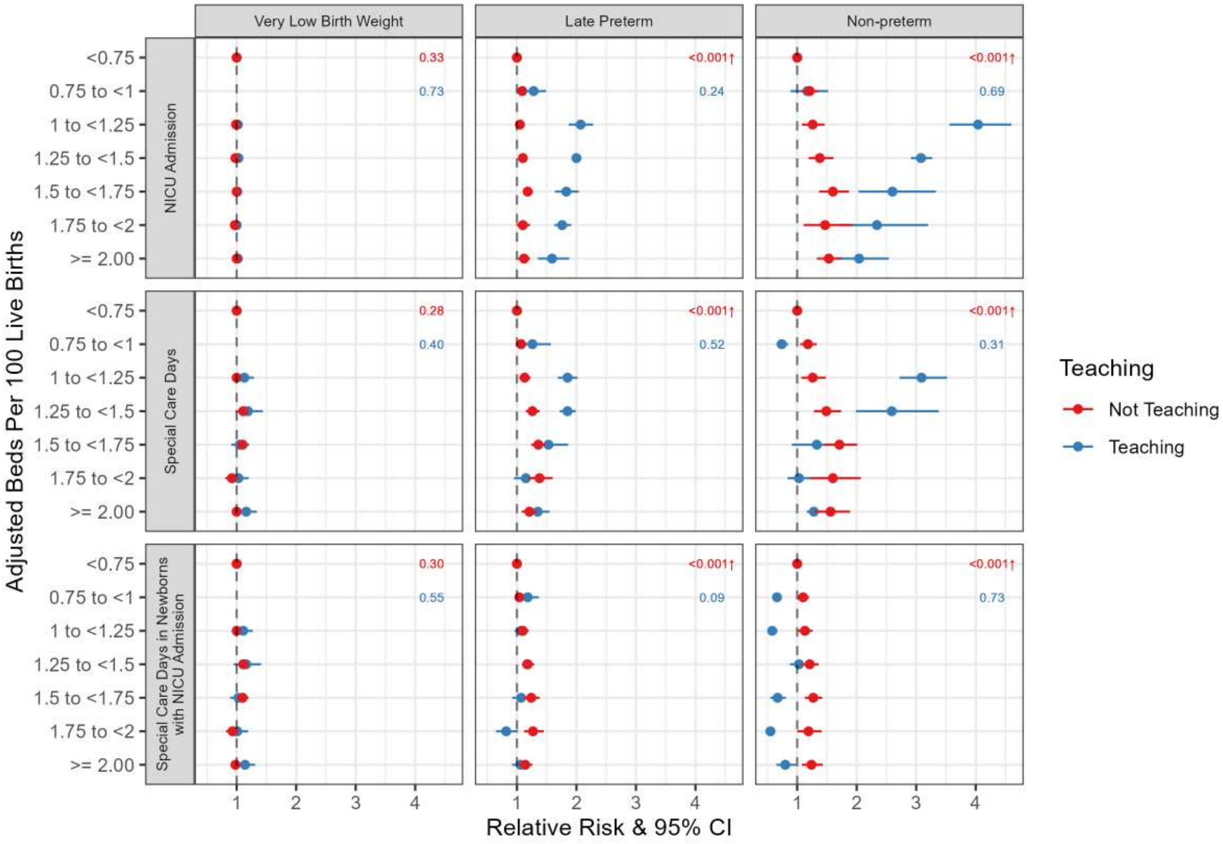

© 2024 Goodman DC et al. *JAMA Network Open.*

eTable 1. Association of Hospital-Level Advanced Care Beds per Live Birth and Inpatient Utilization With All Hospital Nursery Levels, Texas Medicaid, 2010-2014

	Adjusted Risk Ratios (95% Confidence Intervals)								
	Very Low Birth Weight (<1500 gm)			Late Preterm (34-36 week gestation)			Non-preterm (≥ 37 week gestation)		
**NICU beds per 100 live births**									
	**Neonatal Intensive Care Admission**								
<0.5	1			1			1		
0.5 to <0.75	1.50	1.33	1.70	2.06	1.77	2.40	2.32	1.94	2.78
0.75 to <1	1.54	1.36	1.73	2.08	1.78	2.43	2.45	2.02	2.97
1 to <1.25	1.52	1.34	1.71	2.24	1.91	2.63	3.27	2.64	4.05
1.25 to <1.5	1.49	1.32	1.68	2.22	1.90	2.60	3.07	2.51	3.76
1.5 to <1.75	1.52	1.34	1.71	2.37	2.03	2.77	3.46	2.81	4.26
1.75 to <2	1.48	1.31	1.68	2.26	1.91	2.66	3.13	2.37	4.13
>= 2.00	1.53	1.36	1.74	2.17	1.82	2.59	2.93	2.28	3.77
P for trend[a]	<0.001↑			<0.001↑			<0.001↑		
	**Number of Special Care Days**								
<0.5	1			1			1		
0.5 to <0.75	1.01	.93	1.10	1.09	1.01	1.17	1.04	.95	1.15
0.75 to <1	1.00	.91	1.08	1.15	1.05	1.25	1.11	.96	1.27
1 to <1.25	1.02	.94	1.10	1.32	1.21	1.45	1.76	1.45	2.13
1.25 to <1.5	1.11	1.01	1.21	1.38	1.26	1.51	1.59	1.37	1.84
1.5 to <1.75	1.06	.97	1.15	1.44	1.31	1.59	1.60	1.32	1.96
1.75 to <2	.94	.85	1.05	1.28	1.11	1.48	1.28	.97	1.70
>= 2.00	1.02	.94	1.12	1.26	1.14	1.40	1.36	1.11	1.67
P for trend	0.45			<0.001↑			<0.001↑		
	**Number of Special Care Days for Newborns Admitted to a Neonatal Intensive Care Unit**								
<0.5	1			1			1		
0.5 to <0.75	1.05	.95	1.16	.96	.87	1.05	1.06	.95	1.19
0.75 to <1	1.03	.94	1.14	1.04	.95	1.14	1.13	1.02	1.25
1 to <1.25	1.05	.96	1.15	1.07	.96	1.18	1.11	.99	1.26
1.25 to <1.5	1.15	1.04	1.27	1.15	1.04	1.27	1.26	1.10	1.43
1.5 to <1.75	1.10	.99	1.22	1.18	1.06	1.32	1.27	1.12	1.43
1.75 to <2	.98	.87	1.10	1.05	.90	1.22	1.10	.94	1.28
>= 2.00	1.05	.95	1.17	1.11	.99	1.24	1.33	1.15	1.55
P for trend	0.37			<0.001↑			<0.001↑		

Notes:
a. p-value and direction of association

© 2024 Goodman DC et al. *JAMA Network Open.*

eTable 2. Association of Hospital-Level Advanced Care Beds per Live Birth and Inpatient Utilization With Hospital Nursery Levels III and IV, Texas Medicaid, 2010-2014

	Adjusted risk Ratios (95% Confidence Intervals)								
	Very Low Birth Weight (<1500 gm)			Late Preterm (34-36 week gestation)			Non-preterm (≥ 37 week gestation)		
**NICU beds per 100 live births**									
	**Neonatal Intensive Care Admission**								
<0.5	1			1			1		
0.5 to <0.75	1.01	.98	1.04	1.10	.97	1.24	1.25	1.03	1.51
0.75 to <1	1.00	.97	1.03	1.04	.92	1.19	1.18	.96	1.46
1 to <1.25	.98	.95	1.01	1.13	.99	1.29	1.59	1.27	1.99
1.25 to <1.5	.96	.93	1.00	1.12	.98	1.28	1.50	1.21	1.86
1.5 to <1.75	.98	.95	1.01	1.19	1.04	1.36	1.68	1.34	2.09
1.75 to <2	.96	.93	1.00	1.14	.99	1.31	1.53	1.15	2.04
>= 2.00	1.00	.97	1.03	1.13	.97	1.31	1.47	1.13	1.92
P for trend[a]	<0.01↓			0.02↑			<0.001↑		
	**Number of Special Care Days**								
<0.5	1			1			1		
0.5 to <0.75	1.07	.95	1.20	1.08	.95	1.24	1.09	.93	1.29
0.75 to <1	1.04	.92	1.17	1.10	.96	1.27	1.12	.92	1.35
1 to <1.25	1.06	.95	1.19	1.29	1.12	1.48	1.80	1.43	2.26
1.25 to <1.5	1.15	1.02	1.30	1.34	1.16	1.54	1.62	1.33	1.96
1.5 to <1.75	1.10	.97	1.25	1.40	1.22	1.62	1.63	1.29	2.06
1.75 to <2	.98	.86	1.13	1.25	1.04	1.50	1.31	.96	1.78
>= 2.00	1.06	.94	1.20	1.25	1.08	1.46	1.41	1.10	1.82
P for trend	0.75			<0.001↑			<0.001↑		
	**Number of Special Care Days for Newborns Admitted to a Neonatal Intensive Care Unit**								
<0.5	1			1			1		
0.5 to <0.75	1.07	.95	1.21	1.00	.87	1.15	1.07	.90	1.26
0.75 to <1	1.05	.93	1.18	1.09	.95	1.24	1.12	.96	1.31
1 to <1.25	1.07	.95	1.20	1.11	.97	1.28	1.11	.94	1.31
1.25 to <1.5	1.17	1.04	1.32	1.20	1.04	1.38	1.25	1.05	1.49
1.5 to <1.75	1.11	.98	1.26	1.23	1.07	1.43	1.26	1.06	1.49
1.75 to <2	.99	.86	1.13	1.10	.92	1.32	1.10	.90	1.33
>= 2.00	1.07	.94	1.21	1.16	.99	1.35	1.33	1.09	1.61
P for trend	0.63			<0.001↑			<0.001↑		

a. p-value and direction of association

© 2024 Goodman DC et al. *JAMA Network Open.*

eTable 3. Association of NICU Beds per Live Births and Newborn Adverse Events With All Hospital Nursery Levels, Texas Medicaid, 2010-2014

	Adjusted Risk Ratios (95% Confidence Intervals)[a]								
	Very Low Birth Weight (<1500 gm)			Late Preterm (34-36 week gestation)			Non-preterm (≥ 37 week gestation)		
**NICU bed per 100 live births**									
	**Inpatient Mortality**								
<0.5	1			1			1		
0.5 to <0.75	0.86	0.73	1.02	0.96	0.66	1.39	0.61	0.43	0.87
0.75 to <1	0.86	0.73	1.02	0.80	0.52	1.22	0.57	0.39	0.85
1 to <1.25	0.91	0.77	1.09	0.78	0.47	1.31	1.05	0.68	1.61
1.25 to <1.5	0.72	0.60	0.85	1.32	0.91	1.91	1.14	0.70	1.84
1.5 to <1.75	0.79	0.64	0.97	1.07	0.71	1.61	1.29	0.89	1.86
1.75 to <2	0.74	0.59	0.92	1.01	0.62	1.64	1.17	0.76	1.81
>= 2.00	0.94	0.77	1.14	1.39	0.88	2.20	1.63	1.12	2.36
P for trend[b]	0.07			0.06↑			<0.001↑		
	**30-Day Post-discharge Adverse Events**								
<0.5	1			1			1		
0.5 to <0.75	0.93	0.77	1.12	0.95	0.85	1.06	0.91	0.83	1.00
0.75 to <1	0.93	0.75	1.14	1.08	0.97	1.21	1.01	0.93	1.10
1 to <1.25	0.87	0.70	1.08	1.00	0.87	1.15	1.00	0.88	1.14
1.25 to <1.5	0.97	0.80	1.17	1.05	0.94	1.17	1.12	1.00	1.25
1.5 to <1.75	0.96	0.78	1.19	1.14	1.00	1.30	1.18	1.02	1.37
1.75 to <2	1.03	0.79	1.36	1.34	1.14	1.57	1.41	1.15	1.73
>= 2.00	0.82	0.64	1.05	1.09	0.89	1.34	1.13	0.90	1.43
`	0.8			<0.01↑			<0.001↑		

[a] Poisson GEE models were newborn covariates predicted inpatient mortality categories, diagnoses, procedures, congenital anomalies; hospital covariates were volume, profit status, and presence of neonatal fellowship. Adjusted NPT models would not converge, so predicted mortality was specified as a continuous variable. Baseline rates for inpatient mortality were 12.33% (VLBW), 0.42% (LPT), and 0.05% (NPT). Baseline rates for 30-day post-discharge adverse events were 14.10% (VLBW), 9.30% (LPT), and 6.70% (NPT).

[b] p-value and direction of association

© 2024 Goodman DC et al. *JAMA Network Open.*

eTable 4. Association of NICU Beds per Live Births and Newborn Adverse Events With Hospital Nursery Levels III and IV, Texas Medicaid, 2010-2014

	Adjusted Risk Ratios (95% Confidence Intervals)[a]								
	Very Low Birth Weight (<1500 gm)			Late Preterm (34-36 week gestation)			Non-preterm (≥ 37 week gestation)		
**NICU beds per 100 live births**									
	**Inpatient Mortality**								
<0.5	1			1			1		
0.5 to <0.75	0.87	0.71	1.08	0.94	0.53	1.65	0.76	0.44	1.31
0.75 to <1	0.86	0.7	1.05	0.78	0.43	1.41	0.68	0.39	1.19
1 to <1.25	0.92	0.75	1.13	0.73	0.37	1.44	1.2	0.67	2.15
1.25 to <1.5	0.72	0.58	0.88	1.2	0.68	2.1	1.33	0.73	2.43
1.5 to <1.75	0.79	0.63	1	0.99	0.55	1.79	1.48	0.86	2.53
1.75 to <2	0.74	0.58	0.95	0.94	0.5	1.79	1.35	0.76	2.41
>= 2.00	0.94	0.75	1.18	1.32	0.7	2.48	1.91	1.11	3.31
P for trend[b]	0.24			0.08			<0.001↑		
	**30-Day Post-discharge Adverse Events**								
<0.5	1			1			1		
0.5 to <0.75	0.86	0.61	1.21	0.9	0.74	1.1	0.8	0.67	0.95
0.75 to <1	0.91	0.64	1.29	1.05	0.88	1.26	0.91	0.79	1.06
1 to <1.25	0.84	0.59	1.21	0.99	0.81	1.21	0.93	0.77	1.12
1.25 to <1.5	0.95	0.68	1.34	1.04	0.87	1.25	1.06	0.89	1.25
1.5 to <1.75	0.94	0.66	1.34	1.12	0.92	1.36	1.09	0.9	1.33
1.75 to <2	1.01	0.68	1.5	1.32	1.06	1.63	1.3	1.02	1.64
>= 2.00	0.81	0.56	1.19	1.11	0.86	1.44	1.09	0.83	1.43
P for trend	0.68			<0.001↑			<0.001↑		

[a] Poisson GEE models were newborn covariates predicted inpatient mortality categories, diagnoses, procedures, congenital anomalies; hospital covariates were volume, profit status, and presence of neonatal fellowship. Adjusted NPT models would not converge, so predicted mortality was specified as a continuous variable. Baseline rates for inpatient mortality were 12.12% (VLBW), 0.46% (LPT), and 0.06% (NPT). Baseline rates for 30-day post-discharge adverse events were 14.02% (VLBW), 9.36% (LPT), and 6.68% (NPT).

[b] p-value and direction of association

© 2024 Goodman DC et al. *JAMA Network Open.*
