# Supplementary material for: Hospital-Level NICU Capacity, Utilization, and 30-Day Outcomes in Texas
Source: JAMA Netw Open. 2024 Feb 14;7(2):e2355982. doi: 10.1001/jamanetworkopen.2023.55982 (PMC10867701; doi:10.1001/jamanetworkopen.2023.55982)
Supplement: Supplement 2. — Nonauthor Collaborators [file jamanetwopen-e2355982-s002.pdf]

Supplemental Online Content: Nonauthor Collaborators
*First name, last name, and suffix (if applicable) are required and will appear in PubMed.

***Group Name(s): The Texas Neonatal Care Research Collaborative**

***First Name and Middle Initial(s)**	***Last Name**	***Suffix (eg, Jr, III)**	Academic Degrees	Institution	Location (city, state/province, country)	Role or Contribution, eg, chair, principal investigator	Group (if more than 1 Group listed in the byline) and/or Subgroup (eg, Steering Committee)
Kristen K	Bronner		MA	The Dartmouth Institute for Health Policy and Clinical Practice, Geisel School of Medicine at Dartmouth	Lebanon, NH USA	Editor	
Youngran	Kim		PhD	University of Texas, School of Public Health	Houston, TX USA	Investigator	
George	Little		MD	The Department of Pediatrics, Geisel School of Medicine at Dartmouth, Children's Hospital at Dartmouth	Lebanon, NH USA	Investigator	
Joseph	Schulman		MD MS	California Department of Health Care Services	Sacramento, CA USA	Investigator	
Jordan	Taylor		BS	The Dartmouth Institute for Health Policy and Clinical Practice, Geisel School of Medicine at Dartmouth	Lebanon, NH USA	Research project coordinator	
