# Supplementary material for: Hospital-Level NICU Capacity, Utilization, and 30-Day Outcomes in Texas
Source: JAMA Netw Open. 2024 Feb 14;7(2):e2355982. doi: 10.1001/jamanetworkopen.2023.55982 (PMC10867701; doi:10.1001/jamanetworkopen.2023.55982)
Supplement: Supplement 3. — Data Sharing Statement [file jamanetwopen-e2355982-s003.pdf]

Data Sharing Statement

Goodman. Hospital-Level NICU Capacity, Utilization, and 30-Day Outcomes in Texas. *JAMA Netw Open*. Published February 14, 2024. doi:10.1001/jamanetworkopen.2023.55982

Data
Data available: No

Additional Information
Explanation for why data not available: Our data source, the State of Texas Health and Hunan Services, will not allow us to share the data. Researchers can ask the State of Texas for their own access.
